# Understanding the perspectives of recruiters is key to improving randomised controlled trial enrolment: a qualitative evidence synthesis

**DOI:** 10.1186/s13063-022-06818-4

**Published:** 2022-10-20

**Authors:** Nicola Farrar, Daisy Elliott, Catherine Houghton, Marcus Jepson, Nicola Mills, Sangeetha Paramasivan, Lucy Plumb, Julia Wade, Bridget Young, Jenny L. Donovan, Leila Rooshenas

**Affiliations:** 1 Population Health Sciences, University of Bristol, Canynge Hall, 39 Whatley Road, Bristol, BS8 2PS UK; 2 School of Nursing and Midwifery, Áras Moyola, National University of Ireland Galway, Galway, Ireland; 3 UK Kidney Association, UK Renal Registry, Bristol, UK; 4 Department of Public Health, Policy and Systems, Institute of Population Health, University of Liverpool, Liverpool, L69 3GB UK

**Keywords:** Qualitative evidence synthesis, Recruitment, Randomised controlled trials

## Abstract

**Background:**

Recruiting patients to randomised controlled trials (RCTs) is often reported to be challenging, and the evidence base for effective interventions that could be used by staff (recruiters) undertaking recruitment is lacking. Although the experiences and perspectives of recruiters have been widely reported, an evidence synthesis is required in order to inform the development of future interventions. This paper aims to address this by systematically searching and synthesising the evidence on recruiters’ perspectives and experiences of recruiting patients into RCTs.

**Methods:**

A qualitative evidence synthesis (QES) following Thomas and Harden’s approach to thematic synthesis was conducted. The Ovid MEDLINE, CINAHL, EMBASE, PsycInfo, Cochrane Central Register of Controlled Trials, ORRCA and Web of Science electronic databases were searched. Studies were sampled to ensure that the focus of the research was aligned with the phenomena of interest of the QES, their methodological relevance to the QES question, and to include variation across the clinical areas of the studies. The GRADE CERQual framework was used to assess confidence in the review findings.

**Results:**

In total, 9316 studies were identified for screening, which resulted in 128 eligible papers. The application of the QES sampling strategy resulted in 30 papers being included in the final analysis. Five overlapping themes were identified which highlighted the complex manner in which recruiters experience RCT recruitment: (1) recruiting to RCTs in a clinical environment, (2) enthusiasm for the RCT, (3) making judgements about whether to approach a patient, (4) communication challenges, (5) interplay between recruiter and professional roles.

**Conclusions:**

This QES identified factors which contribute to the complexities that recruiters can face in day-to-day clinical settings, and the influence recruiters and non-recruiting healthcare professionals have on opportunities afforded to patients for RCT participation. It has reinforced the importance of considering the clinical setting in its entirety when planning future RCTs and indicated the need to better normalise and support research if it is to become part of day-to-day practice.

**Trial registration:**

PROSPERO CRD42020141297 (registered 11/02/2020).

**Supplementary Information:**

The online version contains supplementary material available at 10.1186/s13063-022-06818-4.

## Background

Recruitment to randomised controlled trials (RCTs) is widely agreed to be challenging, and improving recruitment has been identified as a priority [1]. Research has shown that RCTs often fail to recruit patients ‘to time and target’ [2, 3], with well-documented consequences. Failed recruitment can delay the introduction of promising interventions and perpetuate use of those that are less appropriate than previously assumed. Under-recruitment can also lead to wasted resources when RCTs are discontinued and not reported [4], particularly as the costs required to run RCTs are extensive, often involving collaborations between multiple institutions. Discontinuing RCTs can also have important ethical implications for patients who have enrolled [5], with potential to cause distress and frustration around the failure to attain what the study had set out to achieve. Although there are now many proposed interventions to improve recruitment, a 2018 Cochrane review concluded that there was a need for more evidence in this area, as few interventions were supported by high-certainty evidence according to the Grading of Recommendations Assessment, Development and Evaluation (GRADE) system [6, 7]. Building on the recommendations of this review, a James Lind priority setting exercise identified the top ten uncertainties around trial recruitment research as part of the Prioritising Recruitment in Randomised Trials Priority Setting Partnership (PRioRiTy PSP) study [8]. Subsequent work identified that qualitative methods were appropriate to address the questions identified by the PRioRiTy PSP, highlighting that qualitative evidence synthesis (QES) was an emerging method of bringing together primary qualitative research that could inform both policy and practice [9].

Several of the uncertainties identified by the PRioRiTy PSP related to the ways in which information is communicated to members of the public who are invited to take part in RCTs, as well as the barriers and enablers for clinicians in conducting RCTs [8]. Clinicians are increasingly taking responsibility for recruiting patients to RCTs as part of their day-to-day practice [10] and patients’ decisions about RCT participation are often shaped by healthcare professionals, in part due to the trust that patients have in their clinicians [11]. A review of interventions to improve clinicians’ recruitment activity recommended that understanding and communicating RCT methods should be a target for future interventions for clinicians [12]. Subsequent research has identified that clinicians often find it challenging to communicate the concept of equipoise to patients—something that is key for successful recruitment [10].

The overarching topic of recruiters’ perspectives and experiences of recruiting to RCTs has, however, not yet been synthesised across clinical areas. A 2020 Cochrane review of potential participants’ views and experiences of the RCT recruitment process called for a qualitative evidence synthesis to explore this topic [11]. The aim of this QES was to address the identified gap in the evidence by systematically searching and synthesising the findings on recruiters’ perspectives and experiences of recruiting patients into RCTs.

## Methods

The protocol for this QES was published [13] and registered on PROSPERO (reference CRD42020141297). The ENTREQ statement [14] guided the reporting of the QES (Additional File 1). One change was made to the protocol following publication. Originally, a sensitivity analysis had been planned to assess the adequacy of the data, but in order to align with the GRADE CERQual process of assessing, the level of confidence can be placed in the research findings, published guidance on assessing adequacy was followed [15]. This involved assessing the adequacy of the data in reports by considering both their richness and quantity [15].

### Search strategy

The SPIDER (Sample, Phenomena of Interest, Design, Evaluation and Research Type) tool is an method of defining a qualitative research question and generating search terms [16]. It was adapted for the aims of this research by including an additional field of ‘comparisons’ and was applied to develop the search strategy (Table 1).

**Table 1:** SPIDER search strategy outline

Sample (S)	Studies in which participants included recruiters with a role in approaching potential participants (e.g. patients, carers or parents) to take part in a healthcare-related RCTs. Recruiters included both professionally registered (e.g. doctors, surgeons, physiotherapists, nurses, radiographers, GPs) and non-professionally registered (e.g. clinical trials assistants, research practitioners) members of staff
Phenomena of interest (Pi)	The phenomenon of interest in this study was recruitment to RCTs. Studies which considered recruitment alongside other trial-related activities could be included as long as the recruitment element was clearly reported and distinguishable from other trial-related activities, in so far as findings could be extracted for inclusion in analysis
Design (D)	Primary research that used qualitative approaches/designs to investigate recruiters’ views, experiences and practices/behaviour related to attempts recruiting participants into RCTs could be included. No limits on qualitative theoretical frameworks were applied. Qualitative methods of data collection included, but were not limited to, qualitative interviews (in-depth, unstructured, semi-structured and structured), focus groups and observations (participant/non-participant). The review focused on studies that reported on recruitment to particular types of RCTs: those based in the healthcare sector, and those that randomised at the individual patient (or proxy) level. Studies reporting on cluster randomised trials were not included because they were considered likely to face different challenges than RCTs that randomise at the individual level. Studies that reported results from non-human, non-healthcare, or lab-based RCTs were excluded. The definition of RCTs included pilot or feasibility studies, provided that they were randomised. Qualitative studies of recruitment to hypothetical RCTs were excluded
Evaluation (E)	This synthesis explored the subjective constructs of attitudes, experiences and practices/behaviour of those recruiting patients to RCTs
Research type (R)	The search focused primarily on qualitative research, although mixed methods research was considered for inclusion where the qualitative component was clearly defined and reported
Comparisons	It was considered likely that there would be primary reports from a range of RCTs. We therefore wanted to be able to make comparisons across specialty or clinical field (e.g. oncology and radiology), type of recruiter (e.g. registered and non-registered, nurse and doctor), level of care (e.g. primary, secondary, community) and nature of RCT treatment arms (e.g. standard care vs novel treatment, or less/no treatment). These comparisons were intended to allow for a greater level of examination of some of the intricacies of recruiting to RCTs and to yield insights that were generalisable across RCTs

This review employed a systematic search of the online literature, focusing on electronic databases. Searches were run on 17th July 2019. Databases searched included the following: Ovid MEDLINE, CINAHL, EMBASE, PsycInfo, Cochrane Central Register of Controlled Trials, ORRCA and Web of Science. Although ‘grey literature’, references of the studies included and citation searches of the journal articles were searched using sources such as OpenGrey, Scopus and Google Scholar, no additional studies were identified for inclusion from these sources. Due to limitations in time and resource, only literature reported in English was included. No limits in terms of geography or time since publication were applied to the search.

### Screening

**Fig. 1:**
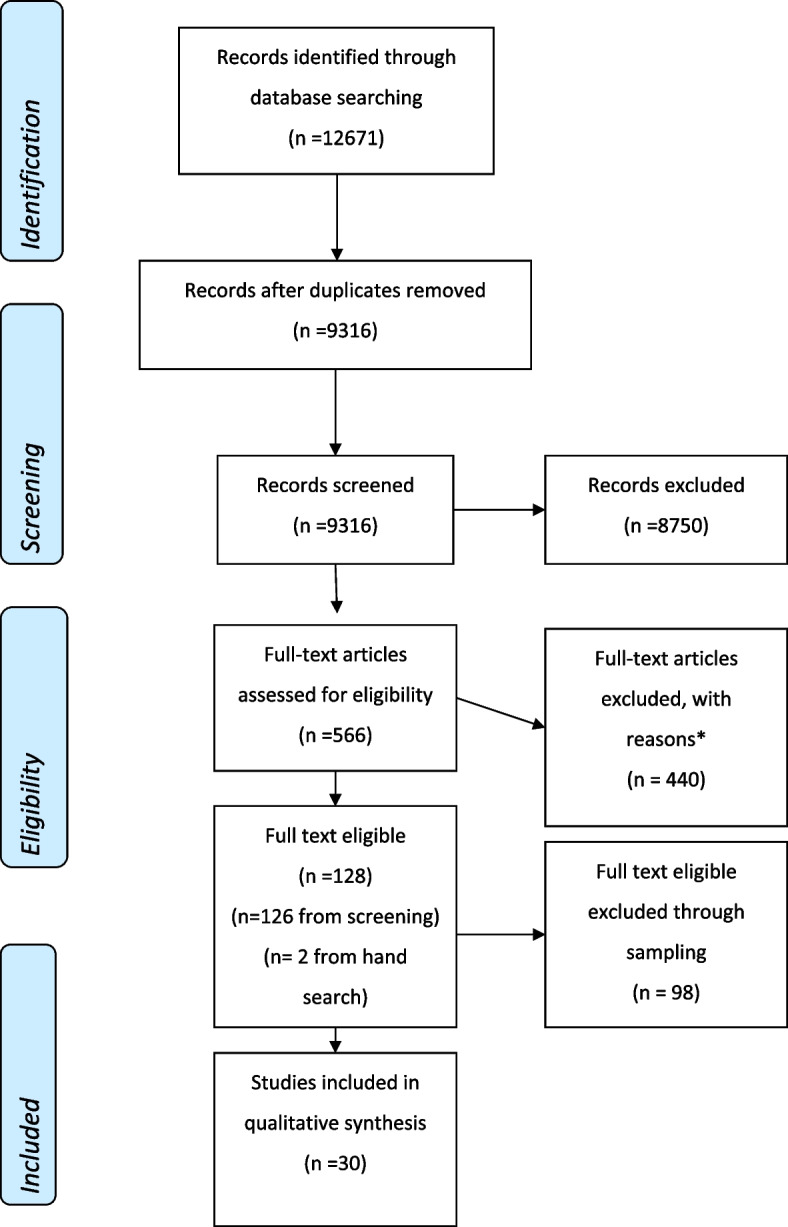
PRISMA diagram of study inclusion process. * Not a study using qualitative methods to explore recruiter perspectives of recruiting patients to an RCT = 231, Study protocol = 5, Cluster RCT = 19, Conference proceedings = 108, Qualitative research was undertaken to explore the intervention, not recruitment = 27, Could not find after repeated attempts = 4, Accidental duplicate = 1, Not in English = 3, Review (not primary research) = 42

All references were imported into EndNote X9, where duplicates were removed. From there, references were imported into Microsoft Excel for a two-stage screening process. Firstly, titles and abstracts were screened by the primary reviewer (NF), whilst a second reviewer (LP) screened 933 of the 9316 (10%) references, which were chosen at random. Due to resource limitations, references without abstracts were excluded at this stage. Different decisions had been made on 43 of the 933 references, which were resolved following discussion between reviewers. A total of 566 papers were retrieved and read for full text review. These 566 papers were considered against the screening tool [Additional File 2] devised for this project which included inclusion and exclusion criteria, and reasons for non-inclusion. The second reviewer also full text screened 5% of these studies. There were no disagreements as to the eligibility of the studies chosen for this review. A total of 126 studies were identified as being eligible for inclusion following this stage of screening, with the authors becoming aware of 2 papers through social media. The screening process, represented using a PRISMA diagram [17], is reported in Fig. 1. Key contextual details were extracted from all 128 eligible papers and stored in Microsoft Excel to aid the subsequent sampling process, which included the fields outlined in Table 2.

**Table 2:** Data extraction for study characteristics and context

Source	Publishing characteristics	Qualitative characteristics	RCT characteristics	Recruiter characteristics	Personal reflections
•Each paper included in the search was assigned a unique number. This was recorded, along with whether the paper had been an initial ‘yes’ or ‘maybe’	•Author (first) •Publication date •Journal •Country of study	•Data collection method(s) •Data analysis method(s) •Sample size •Data collection period	•Number of RCTs from which data is drawn •Healthcare setting •Adult or paediatric •Intervention(s) •Comparator(s)	•Interviewee profession •Experience of recruiters in recruiting (as quantified by authors, often number of patients recruited or years of recruitment experience)	•E.g. whether the findings had already been reported elsewhere, or if anything key was missing (e.g. number of participants)

### Sampling

Qualitative syntheses aim to produce rich, in-depth findings, and it has been proposed that including a very large sample risks jeopardising the researcher’s ability to engage with each individual paper at an analytical level [18]. The contrast between seeking to identify all relevant studies in a traditional systematic review and seeking conceptually rich studies in a QES has been noted in the literature [19]. As such, the review team applied a sampling framework to the 128 papers eligible for inclusion, after agreeing the final sample should be manageable within the scope of a thematic synthesis. The development of the sampling framework was informed by the work of Suri [20], which built on the purposeful sampling techniques described by Patton [21] by extending and adapting these principles to qualitative evidence synthesis. The sampling framework included three main elements, which were considered in parallel. Intensity sampling was used to prioritise the studies where the focus of the research was either solely or in a major way aligned with the phenomena of interest of the QES, exploring the act of recruiting to RCTs, to select the studies where the data were richest. This process was used as several eligible studies explored both recruiting to RCTs, but also evaluating the study intervention. Studies were assessed for their methodological relevance to the QES question using criterion sampling. This involved assessing the extent to which qualitative methods were used (all had qualitative elements as determined by the eligibility criteria, but some included mixed methods research), the extent to which the data reflected the views of staff (as several studies included both staff and patient/proxy views), and the extent to which the reviewers were confident that the staff were recruiting to RCTs (several studies reported mixed recruiter/non-recruiter findings). Maximum variation sampling was applied to ensure that studies from a variety of settings were included, given the agreed broad scope of the review in terms of participant type and setting. Sampling was an iterative process, with the final sample reflecting both what the review team believed to be the best fit for the research question and sampling framework, as well as what would be manageable within the scope of a thematic synthesis. References of studies not included in the final sample are available in Additional File 3.

### Data extraction

As qualitative findings can be reported outside of the typical ‘results’ headings, data were extracted from any section of the paper that they were found in. Data included both verbatim quotes from the study findings, and the interpretations of the study authors.

### Assessing the methodological limitations of included studies

The Critical Appraisal Skills Program (CASP) tool [22], as endorsed by the Cochrane Collaboration [23], was used to appraise the quality of studies included in this review. Application of the CASP was initially piloted on three studies and later applied to all sampled studies by four members of the review team (NF, NM, SP, JW). No studies were excluded as a result of this assessment, but results were documented (see Additional File 4) and used to inform the assessment of the confidence in the review findings.

### Data analysis and synthesis

A thematic synthesis approach, as outlined by Thomas and Harden [19], was used to analyse and synthesise the extracted data. The INTEGRATE-HTA guidance [24] and the subsequently produced RETREAT framework [25] for selecting review methodologies were followed and confirmed the appropriateness of undertaking a thematic synthesis. As advocated by Thomas and Harden, a ‘line-by-line’ approach to analysis was taken by the review team, which initially involved applying descriptive codes to each study individually, by hand. At this stage, the second reviewer (LP) also extracted data and double-coded 10% (3 papers) of the final sample. This provided the opportunity for early emerging concepts to be discussed between members of the team who were both engaged with the literature at an in-depth level.

Second, a stage of ‘translation’ was undertaken, where studies were imported into QSR NVivo (V.12) and codes which had been previously identified were grouped when they captured similar interpretations from different studies. This was the starting point for the development of themes. Finally, we moved to the stage of generating analytical themes to ‘go beyond’ describing the original content. Mind maps were used to consider how the codes were linked by grouping codes which appeared to reflect a similar phenomenon, with review authors contemplating the depth and nuances of each theme in regular analysis meetings (NF, LR, MJ, DE). These meetings helped to ensure themes were representative of the data, with frequent comparisons of NVivo codes and the primary data.

### Assessing confidence in review findings—GRADE CERQual

We followed the GRADE CERQual (Confidence in the Evidence from Reviews of Qualitative research) approach to assess the degree of confidence we could have in the review findings [26]. Following the four-stage approach, we were guided by GRADE CERQual implementation papers [15, 27–30] to consider the four components of the approach: methodological limitations, coherence, adequacy of the data and relevance. Together, these components contribute towards an overall assessment of confidence in the review findings. The primary reviewer (NF) completed the assessment, with input from members of the review team, and reported a judgement about the overall confidence in the evidence supporting each review finding. Confidence in all review findings was initially determined to be high, and where there were concerns about any of the components, the findings were then downgraded accordingly [27]. Classifications were then discussed and agreed amongst the review team. Overall, we had high confidence in the majority of our findings, reporting only one finding in which we had low confidence, due to the concerns about coherence and adequacy. The summary of qualitative findings is reported in Additional File 5, and the detailed full evidence profile is available in Additional File 6.

## Results

Following the sampling process outlined above, 30 studies were included in this synthesis (as outlined in the PRISMA diagram, Fig. 1). Characteristics of the included studies are outlined in Additional File 7. Included studies were published between 2000 and 2019. The majority (*n* = 23, 77%) of studies were from the UK [31–53]. Other countries of study included Canada [54], Australia [55, 56], Sweden [57, 58] and the USA [59, 60]. Studies were mainly described as being in primary [31, 33, 34, 39, 43, 47, 51, 57, 59] or secondary care settings [31–38, 42, 44–46, 48–50, 52–54], but also in palliative [58, 60], psychosis services [41], pharmacy [56], local health district [55] and community care [40] settings. Both registered and non-registered healthcare professionals were reported as those involved in recruitment with the views of several professions represented across the studies. Recruiter roles are reported in Additional File 8.

Five main themes were generated from the synthesis: ‘recruiting to RCTs in a clinical environment’; ‘enthusiasm for the RCT’; ‘making judgements about whether to approach a patient’; ‘communication challenges’ and ‘interplay between recruiter and professional roles’. These themes are often interlinked, highlighting the ways in which structures, values and perceived roles can shape a recruiters’ experience. These themes are described below and illustrated with quotes from the primary studies.

### Theme 1: Recruiting to RCTs in a clinical environment

The process of identifying eligible patients in a clinical environment was reported in many studies as being challenging for several reasons [31, 32, 34, 39, 41, 44, 45, 48, 49, 51, 56–58, 60]. Study authors reported that the way patients engaged with or accessed services could impact on whether patients could be identified or approached for the RCT [32, 39, 44, 49, 58], for example reflecting that teenagers with ‘visible differences’ [p.544] rarely presented in primary care, where recruitment to the YP Face IT study was taking place [39]. When the study design required patient identification through record searches, recruiters from several studies reported difficulties when data were insufficient to make decisions about eligibility [32, 41, 60], because records were out of date or not fit for purpose. Recruiters often reflected that there were fewer patients who were eligible for the RCT than had been anticipated [31, 34, 44, 45, 49]. Some authors [44, 45] attributed this to differences between the anticipated population and the actual eligible population when the study was designed:

*Paleri et al. [*44*] [p.142] (author interpretation):* *“Patient availability, especially in terms of the changes in cancer treatment practices between study design and the TUBE trial implementation, which led to fewer eligible patients who could be approached about recruitment.”*

When recruiting in the clinical environment, some recruiters commented on the practical issues for patients which they believed impacted recruitment [34, 35, 38, 47, 51, 55, 56, 58]. Issues with travelling to clinical appointments or accessing hospitals where treatments would take place were raised as concerns by some recruiters [34, 35, 38, 51]. Although most of these recruiters reported experiencing patients rejecting the RCT due to concerns about travelling or access, Griffin et al. indicated that recruiters themselves lacked confidence to approach patients who did not have easy access to the study site [35]. Others reported practical issues such as patients not being open to being approached about research as they were in a hurry or could not commit the time [55, 56], and potential participants not being present during an attempted recruitment visit [58].

The amount of additional time required for research—especially recruitment processes—was widely discussed by recruiters, with a focus on how already busy workloads could not accommodate the additional requirements of RCTs [32, 37, 39, 41–43, 47, 51, 56–58]. Recruiters in primary care in particular noted how clinic appointments were already stretched and not necessarily extended to accommodate research [39, 43, 57]:

*Mason et al. [*43*] [p.522] (primary quote - GP):* *“It is all down to time. Time, time, time. I think, you know, can you justify doing this and do you have enough time allocated to do it on top of all your other things? Depressed patients take twice as long as other patients that walk through the door, so you’re already up the creek in terms of time. Time-wise, you’re already on a kind of losing wicket because you’ve got this constant fight against the clock in general practice.” (0704 Male)*

Alongside a lack of staff resource, a desire for more research staff time was reported [31, 32, 37, 42, 48, 50, 51, 60]. Recruiters suggested that having additional protected nurse or researcher time would be beneficial for recruitment [32, 42, 48], as was identified by Langley et al. in their study of hospital clinicians:

*Langley et al. [*42*] [p.166] (primary quote – clinician):* *“There’s no doubt that if we had secretarial, nursing support and so forth for running the trial, we would put a lot more people in.”*

Whilst some recruiters who had dedicated staff to undertake research perceived there to be benefits, such as not disrupting routine practice [35], others reported not using the additional research personnel available to them, as they felt it was inappropriate for staff external to the clinical service to be involved in care [41]. A lack of staff support for an RCT and time pressures were often interlinked, with current staff struggling to fulfil their research duties alongside clinical duties.

Within the context of recruiting in the usual clinical environment, recruiters often reported that their and their colleagues’ attitudes towards RCTs in general, or towards a particular RCT, could be problematic for recruitment [31–33, 39, 42–44, 49, 50, 56, 57, 59]. Clinicians often had other competing priorities [39, 43, 49, 56, 57] which were rooted in the belief that the day-to-day care of patients should take precedence, as well as there being a limited emphasis on research amongst the community [32]. In addition, staff feelings of suspicion and distrust towards research [31, 59] were reported, as were concerns around threats to skillsets (such as the ability to practice skills, or the erosion of the need for a particular skillset) [33, 44]. Often these issues were reported as interpretations by the study authors [33, 39, 57, 59], but they were also identified by recruiters as being issues for their colleagues [31, 49, 56] or themselves [32, 43, 44], such as amongst the pharmacist community:

*Shaheed et al. [*56*] [p.989] (primary quote – Pharmacist Recruiter):* *“When first introduced, all pharmacist team members were keen to recruit patients… However, other instore targets superseded the LBP study (not a priority). Many patients have back pain, but often patients [are] already on a treatment, or had tried paracetamol or had a chronic condition.” (Recruiter 13)*

These findings indicate that the attitudes of some involved in recruitment may not be conducive to recruitment, especially when there is perceived to be competition between day-to-day clinical and research priorities.

Where there was a perceived culture of research embedded within practice at sites, this was identified as being beneficial for recruitment [32, 43, 46, 48]. This was in part because a research culture was thought to encourage engagement from the wider team [46, 48]. A ‘research culture’ was not uniformly described, rather related to the concept of research being integrated into day-to-day activity by staff. As an example of how having a research culture was helpful, Phelps et al. reported that when working on several studies, recruiters were willing to identify and approach patients for other research as well as their own studies [46].

### Theme 2: Enthusiasm for the RCT

Several recruiters were enthusiastic about the RCTs they worked on and the prospect of improving the evidence base, either for a specific disease or patients in general [31, 33–39, 42–44, 51–53, 55, 57]. Although, as identified in theme 1, a challenging clinical environment could negatively impact staff attitudes towards recruitment, enthusiasm for the RCT was also beneficial, with recruiters often indicating that they hoped for useful knowledge to be produced from the RCT to guide future practice [31, 34, 36, 44, 51, 52]. Ekambareshwar et al. found that presenting a personal sense of interest and belief in the study when approaching patients aided recruitment [55], and Hallowell et al. highlighted how perceiving the research question to be important and beneficial for patients can benefit recruitment:

*Hallowell et al. [*36*] [p.7] (author interpretation):* *“Got-it was seen as an easy trial to deliver, not only because the intervention is relatively straightforward to administer, but also because the staff identified a pressing clinical need for a drug that would enable women to deliver their placenta safely and simply.”*

Recruiters appeared to hope that by generating knowledge from RCTs the uncertainty they faced when recommending treatments to patients would be reduced [33–35]:

*Griffin et al. [*35*] [p.50] (author interpretation):* *“The majority of these clinicians were enthusiastic about their potential contribution to answering a relevant and valid scientific question through methods they found acceptable.”*

Enthusiasm towards the RCT was also attributed to it addressing a pressing clinical need [36], providing evidence for a potential alternative to a radical treatment [37] and engaging an under-served group [39]. There was also evidence that for some recruiters, their enthusiasm towards the RCT stemmed from a belief in the benefits of the intervention being investigated [31, 47], suggesting a lack of equipoise and a preference for one arm of the RCT:

*Campbell et al. [*31*] [p.41] (author interpretation):* “Some practitioners stated that they explicitly and deliberately recruited to the trial in order to access drugs.”

Support from clinical teams not directly associated with the research was highly valued by recruiters; where clinical staff appeared enthusiastic or there was ‘*buy in*’ [44] [p.65] for the study, it was believed to make the recruitment process easier [31, 36, 44, 46, 50, 54, 60], as it relieved some of the pressures on recruiting staff as Hanson et al. identified in their palliative care research:

*Hanson et al. [*60*] [p.1026] (primary quote – CRC):* *“I think having support from your other team members is extremely helpful and honestly if you have that that would improve recruitment and all of the above.”*

It was, however, often considered difficult to obtain the support of those outside of the research team who may not have shared the same levels of enthusiasm for the study [36, 45, 46, 48, 58]. To achieve a sense of cohesion, recruiters’ enthusiasm for the RCT was essential for integrating the study into the clinical pathway [31, 44, 46, 48], as acknowledged in a urology-based RCT:

*Skea et al. [*48*] [p.4] (primary quote – Research Nurse):* *“I’ll go down to the (department) at 9.00 when the doctors just walk in, just to make sure they’ve got their research heads on as well as their clinical heads and that they will ring us if there’s a patient… So it’s about making them think that research is a normal bit of the hospital, this is the norm as opposed to the exception… we went down… with information given to the registrars and consultants at our monthly meetings and there are posters on the wards. There is a file on every ward where they would get admitted…So getting those nurses engaged…… Yeah, cake usually works, doesn’t it?” (Site 1 RNa)*

### Theme 3: Making judgements about whether to approach a patient

Recruiters suggested several reasons why particular patients may not be approached even if they were eligible according to the RCT protocol and were observed to apply inclusion and exclusion criteria variably, as was identified by Donovan et al. in their cross-trial synthesis [33, 34, 37–41, 43, 45, 46, 49, 53]:

*Donovan et al. [*34*] [p. 5] (primary quote - doctor):* *“My bias is that in a younger person, [intervention 1] probably is a better treatment … Rather than putting them into trial, I think what I’d like to do is give them [intervention 1] up-front.” (T6-D12)*

Reasons for not approaching eligible patients included discomfort about the eligibility criteria [33, 34, 37, 46, 49], assessments of patients’ personal circumstances [34, 53] and fear of burdening patients with the demands inherent in RCT participation (e.g. where one of the RCTs arms might make additional demands on a patient) [40, 43]:

Ziebland et al. [53] [p.4] (author interpretation): *“They talked about psychological and social factors in selecting patients for spinal fusion and the ‘art’, ‘instinct’ and ‘eye’ of the surgeon.”*

Recruiter preferences for particular treatments were key to determining whether an eligible patient was approached [33, 34, 37, 43, 44, 46, 49, 52, 53]. Some recruiters stated intervention preferences, believing in the superiority of that treatment above others [31, 33–35, 37, 38, 40, 44, 45, 53]. Others acknowledged that they had preferences for the treatment of certain individuals or groups of patients with certain characteristics [33, 34, 37, 38, 43–46, 49, 52, 53]. For instance, in one orthopaedic RCT, a recruiting surgeon stated:

*Phelps et al. [*46*] [p.6] (primary quote- surgeon):* *“There are certain patients that would be eligible based on the criteria but whom people are saying no but this obviously needs a plate or no but this obviously needs a nail you would never do the other thing for this fracture. Now I appreciate that this may not be across sites but certainty within this site my perception is that patients are screened eligible but aren’t included because people are going ‘that just shouldn’t have either nail or plate’?” (Surgeon 8)*

Recruiters described how intuition, beliefs, hunches or past experiences influenced their preferences [33–35, 37, 44, 49]. Some preferences were aligned to clinical speciality [33, 44], with recruiters often suggesting that other specialities or groups promoted or believed more in their own treatments [33, 44, 45, 50]. This was illustrated by a recruiting nurse in an oncology RCT:

*Paramasivan et al. [*45*] [p.7] (primary quote – nurse, recruiter):* *“Depends who’s spoken (laughs lightly) to them first, I mean if the surgeons have spoken to them first, they generally think that surgery is the best option because it’s been discussed by a surgeon and to a surgeon that is the best option. If they’ve spoken to an oncologist or myself, if they speak to me, they tend to be more open-minded about it, because I present both sides.” (P6)*

There were instances where individual or team beliefs about what constituted appropriate eligibility criteria misaligned with the actual eligibility criteria or what was described in the protocol [33, 34, 37, 38, 44, 46, 49, 51]. This could lead to discomfort in recruiting patients who were perceived to be on the periphery of what recruiters believed to be clinically appropriate for the study [33, 34, 37, 46, 49], as was identified by Stein et al. in a breast cancer RCT:

*Stein et al. [*49*] [p.180] (author interpretation):* *“Research staff varied in their readiness to accept increasing risk (in terms of disease status). Increasing lymph node involvement, tumour size and grade caused discomfort surrounding the upper thresholds of the eligibility criteria stated in the protocol.”*

This was first proposed by Donovan et al. in their cross-trial synthesis, who suggested that recruiters found it easier to recruit patients when they met a certain ‘core’ set of recruitment criteria [33], rather than being on the ‘edges’ of criteria. In particular, doctors and surgeons were found to struggle to apply the eligibility criteria to particular patients or groups of patients for whom they felt less (un)certain about the best course of treatment, despite them being technically eligible for recruitment [33, 34, 37, 46, 49].

In additional to making clinical judgements, recruiters also described how they would ‘size up’ [54] [p1587] patients by assessing their eligibility beyond the relevant study criteria, including additional factors such as their personality, personal circumstances or motivations to participate [31, 34, 35, 37, 39–47, 49, 51–54]. These factors often helped recruiters to justify their decisions not to approach patients, believing they were acting in the best interests of their patients, as was evidenced in a community care study:

*Howard et al. [*40*] [p.44] (author interpretation):* *“CCs [care coordinators] used their own perceptions of who was ready to work, not necessarily in accordance with each other. This varied from being under motivated due to low self-esteem or over motivated if not yet “ready” for employment.”*

Not approaching patients because they were perceived to be eligible but not suitable was linked by Tomlin et al. to nurses having what they termed as ‘empathic preferences’ for trial arms, which were based on ‘intimate knowledge’ of the patients [52] [p.674]. Non-recruiting clinical staff were also reported by recruiters to act as gate-keepers, sometimes preventing their patients from being asked about participation [32, 41, 50, 54, 60]:

*Sin et al. [*41*] [p.701] (primary quote – CSO):* *“Well they (the clinicians) make a judgement on their (service users’) behalf. It’s the same with, not just yours but with other studies, as soon as they start discussing their patients there are “ifs” and “buts”, ‘oh I don’t know about this because at the moment I don’t think they’re settled’; they’ve just moved to a new house’. They seem trivial little things that seem to get in the way as to why they wouldn’t pass the name over (for further information to be sent).” (Trust 1, CSA D)*

Through subjective application of the eligibility criteria, recruiters and other staff more peripherally involved in recruitment could restrict the pool of patients to approach, thus limiting the total number of eligible patients who could be recruited.

### Theme 4: Communication challenges

Theme 3 identified that there were instances where recruiters’ judgements impacted whether they approached a patient, but there was also evidence they could impact recruiters’ communication practices. When recruiters approached potential participants despite their own preferences, these underlying views about treatments were often acknowledged by recruiters and study authors to potentially shape patient preferences [33, 34, 37, 38, 44, 45, 57]. Some recruiters reported or were observed giving patients direct recommendations, [33, 34, 37, 44] believing that this was the right thing to do, as illustrated by an oncologist recruiter in a head and neck cancer study:

*Paleri et al. [*44*] [p.73] (primary quote – oncologist):* *“Ultimately, we will make clear what our recommendation is because they need guidance. Patients do not necessarily, you know, they are not clinicians. They don’t have the full knowledge as to all the different potential benefits and disadvantages.” (Sally, oncologist, Hamilton)*

Some recruiters acknowledged the impact of the preferences of non-recruiting staff, which could be passed on to patients [38, 44, 45, 49, 50, 57], in particular when patients interacted with these staff earlier in their pathway, prior to the RCT being introduced:

*Hange et al. [*57*] [p.146] (author interpretation):* *“According to the informants, staff members who were negative towards the ICBT [Internet-mediated cognitive behavioural therapy] method influenced the patient’s attitude towards treatment and study participation.”*

The challenges of explaining the study to potential participants were widely discussed [32, 35, 37, 38, 40, 42, 43, 45, 48, 49, 53, 57, 58] and were predominantly identified by recruiters [32, 37, 40, 42, 43, 49, 53, 57, 58] themselves. Some recruiters were reported (or observed) to use problematic terms or inaccurate explanations to describe the study design [35, 37, 38, 45, 49, 53], as was found by Paramasivan et al., who observed that certain terms such as ‘gold standard’ were ‘loaded’ and could influence treatment preferences, and subsequently, recruitment [45]. In particular, the topic of randomisation [32, 35, 37, 40, 42, 48, 49, 58] was identified by recruiters [32, 40, 58] and interpreted by study authors as challenging to explain [35, 37, 42, 48, 49].

A key element of communication about the RCT discussed in studies revolved around recruiters’ responses to patients’ articulated treatment preferences. Several recruiters reported that patients’ own treatment preferences made the process of recruitment more of a challenge [34, 37, 38, 42, 44, 45, 48, 49, 56]. Preferences could be for treatments not included in the RCT, or for one of the treatments in the RCT. These preferences were often accepted by recruiters [34, 37, 38, 45, 48, 49] as they were considered to be reasonable, or not amenable to change:

*Skea et al. [*48*] [p.5] (primary quote – research nurse):* *“…one of the obstacles to recruitment is, that patients do express a preference for one treatment or the other based on their own circumstances… in a way I think that that’s free choice…I think sometimes there’s personal reasons that some people would prefer not to have a general anaesthetic, would prefer not to have to stay in overnight…” (Site 2 RN)*

Some authors suggested that when a patient’s preference aligned with that of the recruiter, these were more readily accepted without further discussion [34, 37]. Some recruiters acknowledged the communication difficulties around responding to preferences, believing that it would not be ethical to do so [34, 45], that the patient’s decision was ‘*sensible*’ [37] [p.67], or ‘*reasonable*’ [38] [p.2335], or because they feared doing so could appear coercive [49].

### Theme 5: Interplay between recruiter and professional roles

Several authors described the interplay between recruiters’ clinical or usual role and the recruiter role [31, 33, 34, 39, 40, 43, 44, 46, 47, 49, 51, 52, 58, 59]. Difficulties approaching eligible patients were discussed above in relation to recruiters’ discomfort about eligibility criteria, but some studies also highlighted that discomfort around approaching eligible patients could also relate to clinicians’ self-perceived identities of being caregivers and patient advocates. Of particular note was the balance between being a recruiter and someone who advocated for and protected the patient [31, 34, 40, 43, 46, 49, 51, 52, 58, 59], which was highlighted particularly by primary care clinicians [31, 39, 43, 57, 59]and was described by a GP who indicated a potential conflict of interest between healthcare and research activities:

*Frayne et al. [*59*] [p.452] (primary quote – physician):* *“We are undermining our own credibility as a primary care providers. We are advocates. Sometimes you feel like you are pushing too hard and starting to transition across the other side.”*

There were a small number of examples of the recruiter role being perceived as easier than their professional role:

*Donovan et al. [*33*] [p.915] (primary quote -doctor ):* *“[In clinical practice] I have to make a decision and in the other I don’t and that’s why I prefer trials … I can hide behind a trial, I don’t have to make a decision… I’m doing the best for the patient because I genuinely don’t know; we’re doing the best for society because hopefully we’ll get that answer out.” (T2-D1)*

For nurses in particular, there was a conflict between the role of recruiter and the caring role traditionally associated with nursing [31, 34, 46, 47, 52]:

*Potter et al. [*47*] [p.445] (author interpretation):* *“There was also evidence of nurses trying to protect patients who they felt may not be suitable for the trial perhaps because of difficult personal circumstances.”*

Both recruiters and study authors identified that discomfort in the recruiter role impacted clinicians’ ability to recruit patients as their primary professional role took precedence [34, 52]. Some nurse recruiters felt that their roles as nurses could not be encroached upon and that they were clinicians ‘*first and foremost*’ [52] [p.673]. Others managed to situate their research role within their more prominent role as nurses [31], suggesting that their identity as a nurse was adaptable but still ultimately the dominant one. Nursing values and experiences were brought to the recruitment interaction, with nurse identities influencing their actions and decisions, rather than simply relying on the protocol [31, 34, 46, 52]. This was often justified by describing their role as that of patient advocate, not just a recruiter, as was identified by Campbell et al. in their multi-study review [31, 34, 52]:

*Campbell et al. [*31*] [p.39] (primary quote – nurse):* *“If a patient were to come to a clinic, and the consultant or doctor [said that] this person would be feasible to go into FOCUS, say if they’d already had chemo in the past, or they might not be fit and well, the medical staff might view it a bit differently to how we would. We might think they’re not suitable to go into a study. I think a lot of the time we’re looking at things from a different perspective, perhaps we’re looking at things more as an advocate. And although we are there to recruit, we’re also there to protect the patients… They [can] be quite a vulnerable group.”*

This review identified that those with recruitment responsibilities, in particular registered professionals such as doctors or nurses, may feel a sense of discomfort when they perceive that their ‘recruiter’ role does not fit with their healthcare professional identities.

## Discussion

This review has synthesised published evidence relating to recruiters’ experiences and perspectives on RCT recruitment, addressing a recognised gap identified by a 2020 Cochrane evidence synthesis [11]. We found that overlapping themes relating to recruiting within a clinical environment, enthusiasm for the RCT, judgement around the eligibility criteria, communication with potential participants and recruiters’ dichotomous/conflicting roles provide insight into why recruitment is challenging and often poor.

Considering the impact that the clinical environment has on recruiters, the synthesis identified that recruiters often operate in an environment which is pressed for both time and resources. A lack of time and funding has been noted previously as a barrier to NHS staff engaging with research [61] and was identified in Newington and Metcalfe’s review of researchers’ and clinicians’ perceptions of recruiting to clinical research [62]. Within the clinical environment, cultures and attitudes towards RCTs were reported to impact recruitment, with other priorities often taking precedence particularly when their primary role was not as a recruiter. In the UK, The Department of Health and Social Care published a policy paper on ‘The Future of UK Clinical Research Delivery’, which highlighted how research is currently not always a priority for everyone—something which the authors argue must change and become part of the day-to-day for NHS staff [63]. Top-down drivers to promote this culture change may help to provide reassurance to recruiters that engagement in research will not deter from their core roles, although the practicalities of how to reconcile research work with clinical work needs further thought. Based on the challenges synthesised in this paper, it appears that system-level change is needed to integrate research into clinical practice, as current clinical setups are often not conducive to integrating research into day-to-day practice. There is current work ongoing to propose practical solutions to reconciling research with clinical practice, with exploration of how RCTs can become part of routine care identified as the top priority for members of the trials community, during a 2016 James Lind Alliance Priority Setting Partnership of trial recruitment uncertainties [8].

Enthusiasm for the RCT and its potential outcomes was identified as being beneficial for recruitment. Buy-in from other clinicians was considered helpful, perhaps because sharing the workload and responsibilities went some way to overcoming the lack of resources that recruiters faced. The importance of teamwork when recruiting to studies has been noted previously, both in respect to teamwork with the clinical and research teams [64]. These findings suggest that the demarcation between clinical and research teams may be unhelpful, and that for research to be truly embedded into a healthcare system, an integrative approach which considers the potential roles and actions of those outside the traditionally considered ‘research team’ would be beneficial. This may require extension of support and training to a wider array of staff within the clinical environment, tailored to their role and involvement with the RCT. Training for those recruiting to RCTs, such as the QuinteT RCT Recruitment Training, has been shown to improve self-confidence in discussing RCTs with potential participants [65, 66], and there may be value in such training for those with roles not directly associated with recruitment.

When making judgements about whether to approach a patient, this review identified that clinicians may feel conflicted between what their clinical experience tells them is best for the patient and what is asked of them in the RCT, which could result in eligible patients not being approached to consider RCT participation. These insights were first proposed by Donovan et al., who found that recruiters often felt more comfortable recruiting and expressing equipoise for patients who they felt fitted a set of ‘core’ [p.914] eligibility criteria, but for those on the outskirts of these criteria, their personal boundaries often impacted whether they would attempt to recruit these patients [33]. Furthermore, a distinction emerged from synthesised studies between recruiters not approaching on the grounds of eligibility discomforts, and failure to approach on the basis of patients’ suitability to take on the prospect of RCT participation, resulting in recruiters acting as ‘gate-keepers’ for patient participation. Hanrahan et al. also identified that gate-keeping was prevalent in their study of recruitment in the obstetric care setting [67]. The gate-keeping actions described in this QES and that of Hanrahan et al. may deny patients the ability to make their own decisions about their healthcare [67], and also impact the inclusion of under-served groups in RCTs. Sharkey et al. argue that gate-keeping by clinicians is not ethically justifiable and suggest how collaboration between clinicians and researchers to design and conduct research may help to overcome some of the reasons why patients are not approached [68]. With an increasing focus on how those recruited to RCTs are often not representative of the population the trial results apply to [69], gate-keeping risks exacerbating the production of evidence that does not meet the needs of those who stand to benefit. Early identification of recruiters’ personal positions of equipoise may be beneficial to recruitment so potential issues can be addressed as early as possible. Prior to recruitment starting, the trial management group could develop ‘case studies’ of hypothetical patients who meet the study inclusion criteria but whom may have characteristics that could cause recruiters discomfort as a way of elucidating concerns prior to recruitment starting. Some trial teams have already undertaken similar preliminary work, using vignettes of example patients as part of training or feedback for recruiters [49, 70]. The exploration of recruiters’ views about eligibility and equipoise, including how these are expressed to patients, is a core component of a QRI [10, 33].

Authors of the synthesised papers, and recruiters themselves, often identified how they, and their non-recruiting colleagues, could influence decisions regarding recruitment through their communication practices. Though not included in the sample synthesised, Rooshenas et al. collated audio-recorded recruitment discussions across six different RCTs to explore how equipoise was conveyed. They found that recruiters can often struggle to convey equipoise when explaining RCTs to patients, with their personal views on the trial treatments influencing their communication practices. The study was the first to identify common ways in which equipoise could be overridden or undermined during consultations [10]. Subsequent research, captured in a synthesis of patients’ experiences of being invited to take part in RCTs, reinforced the influential role HCP recruiters’ communication during consultations, in part owing to the trust that patients place in HCPs [11]. These findings indicate the need for further work to integrate research into clinical pathways and raise awareness amongst clinical colleagues about their own communication practices.

The issues related to the ‘dual role’ of clinician researcher have been well established in the literature [65, 71, 72], with Hay-Smith et al. highlighting how both clinician and patient ‘blueprints’ [pg.12] are brought to the recruitment encounter, meaning the traditional clinician-patient relationship may be observed within the encounter [72]. This was also highlighted in Hanrahan et al.’s evidence synthesis of recruitment specifically to RCTs during pregnancy and childbirth, which found that issues related to the ‘dual role’ of clinicians in recruitment shaped the manner in which recruiters went about the task, prioritising clinical care over recruitment [67]. The synthesis reported here has brought together several studies which highlighted how nurses involved in research can face challenges to their identity and their perceived role as a patient advocate [73]. Doctors too can face threats in relation to perceptions of eligibility and equipoise [33]. It may be beneficial to recognise that different professionals (e.g., doctors and nurses) may have different training requirements based on their roles and the challenges that they face [74]. Whilst the literature frequently highlighted ‘dual’ or opposing roles, a shift in culture towards better integration of research into clinical practice may be conducive to reconciling clinical and research roles. Examples already exist, such as in paediatric oncology, where there is reportedly more harmony between clinical and research roles, with their clinical and research roles being described as ‘interwoven’ [75] [p.3]. Rather than highlighting conflicts and tensions, there are opportunities to identify complementary elements of research and clinical practice, as was found by Hanrahan et al., who identified that recruiters to maternity studies found their clinical experience in maternity care to be beneficial to their recruiter role [76]. Clinical and research roles do not have to be viewed as opposing, nor should we assume that those with ‘dual roles’ would be willing (or able) to separate their two practices [77]. Training that encourages clinicians to be reflexive of their involvement may help ensure the strengths of both roles are utilised appropriately.

Many of the recommendations presented here are focused around providing support and training for those involved in recruitment, or require intervention from the trial management team. Given the issues of clinician time and resource raised in this QES, as well as the cost associated with ‘in-person’ events when RCT budgets are stretched, it is important to take a pragmatic approach to enacting these recommendations. In 2018, a ‘study within a trial’ (SWAT) compared on-site with remote set-up meetings for a surgical RCT and found that remote meetings did not adversely affect the study, including recruitment rates [78]. Moving towards an ‘online first’ approach for training may lower the associated costs and also improve accessibility for participants.

### Strengths and limitations

This review was undertaken with careful consideration to methodological rigour, following key guidance to establish the confidence in the evidence presented in this synthesis [26]. Through the application of GRADE CERQual, we have identified a level of confidence in the findings, having generally high or moderate confidence in each of the findings, and have also highlighted the range of RCT settings in which the findings may be applicable, including both primary and secondary care RCTs in a diverse range of clinical areas. A limitation of the findings is their applicability in paediatric or emergency settings, or where recruitment involves patients who do not have capacity to make a participation decision; further exploration of these areas is warranted. Although these populations were not excluded from analysis, the studies included were prioritised on the basis of relatedness to the phenomena of interest and methodological relevance and did not happen to be in the emergency or paediatric setting. In addition, although no limitations on the search were used in terms of publication date or geography, the purposeful sampling strategy may have excluded relevant studies. It is also possible that potentially eligible papers were missed during the screening stage, although the use of a second reviewer for a portion of the studies will have helped to mitigate this risk.

A limitation of this review was that none of the research included was conducted in lower- or middle-income countries (LMICs). Given the relative lack of evidence of recruiters’ experiences reported using QES methods, it was felt that this initial review should be kept as broad as possible and not look at distinct sub-groups or settings, which may warrant their own, separate synthesis to avoid dilution of important, context-sensitive issues. As such, diversity in country income status was not considered when applying sampling criteria, and the perspectives of recruiters from LMICs were not represented in this synthesis. The synthesis question also made no distinction between who the potential participants to be recruited were. Only one study included in the synthesis [59] specifically focused on the views of those recruiting ‘low income and minority women’. Given that representation of under-served groups (including black, Asian and minority ethnic communities) in RCTs is poor [79], further research that looks specifically at recruiter perspectives and experiences of recruiting under-served groups is crucial to understanding and improving their involvement in RCTs. The broad nature of this review meant it was difficult to draw out many meaningful comparisons, e.g. between recruitment to primary or secondary care RCTs. More focused review questions, or questions centred on particular clinical areas, may be more conducive to comparison across different contexts. It is also important to note that a third of the evidence synthesised in this review in based on the research of the QuinteT group and its recruitment improvement intervention, the QRI [80, 81]. This could be considered both a strength and a limitation, given the findings of this group were based on a number of studies across a range of contexts with RCTs that were challenging for recruitment, the findings of which were reinforced across a number of the studies included in this review. As the QuinteT research programme has focused heavily on recruiters’ practices and perspectives [80], it was perhaps unsurprising that many relevant papers from this research group were included in the final sample, but including studies from a broader range of authors may have enabled the incorporation of more diverse findings. Nonetheless, two thirds of the papers were led by other institutions and groups, and this field continues to grow.

Observations around the inclusion of particular studies raise the question of how sampling is undertaken in qualitative evidence syntheses. As previously identified, unlike in traditional systematic reviews, there is an understanding that a purposeful approach to sampling may be more appropriate than an exhaustive approach for a QES [20]. A consequence of a purposeful approach is that when the synthesis question is broad, not all eligible studies may be included in the review. This can raise challenging methodological questions for review teams about their approach to sampling. Certain purposeful approaches, such as using the ‘CART’ (completeness, accuracy, relevance, timeliness) criteria [82] may lead to seminal studies from which the sampled research stemmed, not being included in the review. When decisions are required based upon subjective criteria, there is scope for differences in interpretation and therefore disagreement over the relevance of the final sample. The input of an experienced team can help reassure those conducting the review that their sample is relevant and appropriate, and concerns must be balanced against the feasibility of conducting a meaningful and in-depth synthesis of a larger number of studies.

Finally, our study team consisted of a diverse group of professionals, including mixed-method and qualitative researchers, both with and without clinical backgrounds, which facilitated discussions throughout the analysis.

## Conclusion

This research has identified inter-related, often complex reasons why recruitment is a challenging process. Pressures of limited time and resources in clinical environments are widespread and may compound the challenges recruiters, in particular clinical recruiters, face in recruiting to RCTs. Several of the themes reported in this synthesis appear interlinked and stem from recruiters’ personal views and beliefs related to the RCT or superiority/inferiority of certain treatments. If recruiters do not feel the RCT is in the best interests of the patient, they can refrain from approaching eligible individuals, or do so in a way which either wittingly or unwittingly conveys their personal beliefs about the appropriateness of a treatment to the patient. When considered alongside the published Cochrane review of potential participants’ perspectives [11], it becomes clearer that healthcare professionals can influence patients’ decisions related to RCT participation. If research is to become part of the ‘day-to-day’ of clinical practice as endorsed by the UK government’s Department of Health and Social Care [63], further research priorities should focus on how the wide array of staff involved in patient care can be supported and trained to ensure research participation is offered to all eligible patients, alongside the development of effective strategies to normalise and successfully integrate research into routine clinical practice. 

## Supplementary Information

**Additional file 1.** ENTREQ. Required reporting statement for synthesis of qualitative research.

**Additional file 2.** Screening Form. Form used to aid reviewers when screening papers for inclusion.

**Additional file 3.** Eligible Not Included Studies. References of studies that were eligible for the synthesis but not included.

**Additional file 4.** CASP. Critical Appraisal Skills Programme for appraising a qualitative study.

**Additional file 5.** SoQF. GRADE CERQual Summary of Qualitative Findings.

**Additional file 6.** Full EP. GRADE CERQual full evidence profile.

**Additional file 7.** Study Characteristics. Characteristics of each of the studies included in the synthesis.

**Additional file 8.** Recruiter Roles. Outline of the roles of the recruiters included in the synthesis.

## Data Availability

All relevant data are provided within this paper and appendices.
