# Supplementary material for: Understanding the perspectives of recruiters is key to improving randomised controlled trial enrolment: a qualitative evidence synthesis
Source: Trials. 2022 Oct 20;23:883. doi: 10.1186/s13063-022-06818-4 (PMC9585862; doi:10.1186/s13063-022-06818-4)
Supplement: Supplementary file 2 — Additional file 2. Screening Form. Form used to aid reviewers when screening papers for inclusion. [file 13063_2022_6818_MOESM2_ESM.pdf]

Understanding the perspectives of recruiters is key to improving randomised controlled trial enrolment: a qualitative evidence synthesis – Additional File 2

Inclusion criteria:

- Ability to extract data explicitly related to the recruitment element of involvement of RCTs
- People with the **responsibility of recruiting participants ("recruiters") (e.g. nurse, surgeon, oncologist, research facilitator)** are the focus of the qualitative research
- The qualitative research explores the recruiters' **perspectives or experiences** of the process of recruiting potential participants
- Evidence that the experience of recruitment is drawn from **direct involvement in recruiting to RCTs**
- Qualitative methods must be used to **collect and analyse** the perspectives or experiences of recruiters

Exclusion criteria:

- Studies focusing on the perspectives/experiences of referring patients to RCTs, with no recruitment influence
- Studies where it is not possible to differentiate the findings that are drawn from experience of recruiting to RCTs and experience of recruiting to more generically defined research
- Protocols
- Conferences proceedings
- Cluster randomised trials
- Not qualitative approach to data collection or analysis
- Evaluations of RCT intervention (e.g. how well do staff perceive the trial intervention working)
- Hypothetical RCTs
- Non-English language
- Systematic reviews

Reasons for exclusion:

1. Not a study using qualitative methods to explore recruiter perspectives of recruiting patients to an RCT
2. Study protocol
3. Cluster randomised RCT
4. Exploring views but not using qualitative methods
5. Conference proceedings/poster/presentation
6. Qualitative research was undertaken to explore the intervention, not recruitment (e.g. acceptability of a behaviour change intervention)
7. Could not find after repeated attempts
8. Accidental duplicate
9. Not in English
10. Review (not primary research)

Reasons for maybes:

1. Unclear as to whether the member of staff who are participating was a recruiter
