# Supplementary material for: Understanding the perspectives of recruiters is key to improving randomised controlled trial enrolment: a qualitative evidence synthesis
Source: Trials. 2022 Oct 20;23:883. doi: 10.1186/s13063-022-06818-4 (PMC9585862; doi:10.1186/s13063-022-06818-4)
Supplement: Supplementary file 3 — Additional file 3. Eligible Not Included Studies. References of studies that were eligible for the synthesis but not included. [file 13063_2022_6818_MOESM3_ESM.pdf]

Understanding the perspectives of recruiters is key to improving randomised controlled trial enrolment: a qualitative evidence synthesis – Additional File 3

References of studies not included in final QES sample

1. A. Keightley JC, A. Maguire, C. Speed and N. Innes. Participant recruitment to FiCTION, a primary dental care trial - survey of facilitators and barriers. British Dental Journal. 2014;217(10):5.
2. Amiel P, Moreau D, Vincent-Genod C, Alberti C, Hankard R, Ravaud P, et al. Noninvitation of eligible individuals to participate in pediatric studies - A qualitative study. Archives of Pediatrics & Adolescent Medicine. 2007;161(5):446-50.
3. Ankolekar S, Parry R, Sprigg N, Siriwardena AN, Bath PMW. Views of Paramedics on Their Role in an Out-of-Hospital Ambulance-Based Trial in Ultra-Acute Stroke: Qualitative Data From the Rapid Intervention With Glyceryl Trinitrate in Hypertensive Stroke Trial (RIGHT). Annals of Emergency Medicine. 2014;64(6):640-8.
4. B. E. Gantschnig IN, A. G. Fisher, C. Kunzle and J. Page. Feasibility study of a single-blind randomised controlled trial of an occupational therapy intervention. Scandinavian Journal of Occupational Therapy. 2016;23(4):260-71.
5. Bampton J, Vargas J, Wu R, Potts S, Lance A, Scrivener K, et al. Clinical physiotherapists had both positive and negative perceptions about delivering two different interventions in a clinical trial: a mixed methods study. Journal of physiotherapy. 2012;58(4):255-60.
6. Benson PR, Roth LH, Winslade WJ. Informed consent in psychiatric research: preliminary findings from an ongoing investigation. Social science & medicine (1982). 1985;20(12):1331-41.
7. Bill-Axelson A, Christensson A, Carlsson M, Norlen BJ, Holmberg L. Experiences of randomization: Interviews with patients and clinicians in the SPCG-IV trial. Scandinavian Journal of Urology and Nephrology. 2008;42(4):358-63.
8. Bird L, Arthur A, Cox K. "Did the trial kill the intervention?" experiences from the development, implementation and evaluation of a complex intervention. BMC medical research methodology. 2011;11:24.
9. Blazeby JM, Strong S, Donovan JL, Wilson C, Hollingworth W, Crosby T, et al. Feasibility RCT of definitive chemoradiotherapy or chemotherapy and surgery for oesophageal squamous cell cancer. British Journal of Cancer. 2014;111(2):234-40.
10. Bonsu JM, Frasso R, Curry AE. Lessons from the field: the conduct of randomized controlled trials in Botswana. Trials. 2017;18:6.
11. Boxall L, Hemsley A, White N. Exploring recruitment issues in stroke research: a qualitative study of nurse researchers' experiences. Nurse Researcher. 2016;23(5):8-14.
12. Burke NJ. Rethinking the therapeutic misconception: social justice, patient advocacy, and cancer clinical trial recruitment in the US safety net. Bmc Medical Ethics. 2014;15:7.
13. Byrne-Davis LMT, Salmon P, Gravenhorst K, Eden TOB, Young B. Balancing high accrual and ethical recruitment in paediatric oncology: a qualitative study of the 'look and feel' of clinical trial discussions. BMC medical research methodology. 2010;10:101.
14. Caldwell PHY, Butow PN, Craig JC. Pediatricians' attitudes toward randomized controlled trials involving children. Journal of Pediatrics. 2002;141(6):798-803.
15. Carlisle K, Warren R. A qualitative case study of telehealth for in-home monitoring to support the management of type 2 diabetes. Journal of Telemedicine and Telecare. 2013;19(7):372-5.
16. Chatterjee S, Kieselbach B, Naik S, Kumar S, John S, Balaji M, et al. Customising informed consent procedures for people with schizophrenia in India. Social Psychiatry and Psychiatric Epidemiology: The International Journal for Research in Social and Genetic Epidemiology and Mental Health Services. 2015;50(10):1527-36.
17. Chhoa CY, Sawyer A, Ayers S, Pushpa-Rajah A, Duley L. Clinicians' views and experiences of offering two alternative consent pathways for participation in a preterm intrapartum trial: a qualitative study. Trials. 2017;18:1-10.

18. Clement C, Rapport F, Seagrove A, Alrubaiy L, Williams J. Healthcare professionals' views of the use and administration of two salvage therapy drugs for acute ulcerative colitis: A nested qualitative study within the CONSTRUCT trial. BMJ Open. 2017;7(2):e014512.
19. Cresswell P, Gilmour J. The informed consent process in randomised controlled trials: a nurse-led process. Nursing praxis in New Zealand inc. 2014;30(1):17-28.
20. D. L. Velott SAB, M. M. Hillemeier and C. S. Weisman. Participant Recruitment to a Randomized Trial of a Community-Based Behavioral Intervention for Pre- and Interconceptional Women. Findings From the Central Pennsylvania Women's Health Study. Women's Health Issues. 2008;18(3):217-24.
21. Do CK, Smith CA, Dahlen H, Bisits A, Schmied V. Moxibustion for cephalic version: a feasibility randomised controlled trial. Bmc Complementary and Alternative Medicine. 2011;11:9.
22. Dohan GJaD. Recruiting minorities where they receive care: Institutional barriers to cancer clinical trials recruitment in a safety-net hospital. Contemporary Clinical Trials. 2009;30(6):552-7.
23. Donovan J, Mills N, Smith M, Brindle L, Jacoby A, Peters T, et al. Quality improvement report - Improving design and conduct of randomised trials by embedding them in qualitative research: ProtecT (prostate testing for cancer and treatment) study. British Medical Journal. 2002;325(7367):766-9.
24. Donovan JL, Rooshenas L, Jepson M, Elliott D, Wade J, Avery K, et al. Optimising recruitment and informed consent in randomised controlled trials: the development and implementation of the Quintet Recruitment Intervention (QRI). Trials. 2016;17:11.
25. Durant RW, Wenzel JA, Scarinci IC, Paterniti DA, Fouad MN, Hurd TC, et al. Perspectives on barriers and facilitators to minority recruitment for clinical trials among cancer center leaders, investigators, research staff, and referring clinicians: enhancing minority participation in clinical trials (EMPaCT). Cancer. 2014;120 Suppl 7:1097-105.
26. E. Crawley NM, L. Beasant, D. Johnson, S. M. Collin, Z. Deans, K. White and A. Montgomery. The feasibility and acceptability of conducting a trial of specialist medical care and the Lightning Process in children with chronic fatigue syndrome: feasibility randomized controlled trial (SMILE study). Trials. 2013;14.
27. Elliott D, Hamdy FC, Leslie TA, Rosario D, Dudderidge T, Hindley R, et al. Overcoming difficulties with equipoise to enable recruitment to a randomised controlled trial of partial ablation vs radical prostatectomy for unilateral localised prostate cancer. Bju International. 2018;122(6):970-7.
28. Ellis J, Warden J, Molassiotis A, Mackereth P, Lloyd-Williams M, Bailey C, et al. Participation in a randomised controlled feasibility study of a complex intervention for the management of the Respiratory Symptom Distress Cluster in lung cancer: patient, carer and research staff views. European journal of cancer care. 2017;26(6).
29. Farley A, Tearne S, Taskila T, Williams RH, MacAskill S, Etter JF, et al. A mixed methods feasibility study of nicotine-assisted smoking reduction programmes delivered by community pharmacists - The RedPharm study. BMC public health. 2017;17(1):210.
30. Ferguson PR. Information giving in clinical trials: the views of medical researchers. Bioethics. 2003;17(1):101-11.
31. Freedman TG. The Breast Cancer Prevention Trial: nurses' observations. Cancer nursing. 1998;21(3):178-86.
32. French C, Stavropoulou C. Specialist nurses' perceptions of inviting patients to participate in clinical research studies: a qualitative descriptive study of barriers and facilitators. BMC medical research methodology. 2016;16(1):96.
33. G. Kaur RLS, C. V. E. Powell and P. Williamson. A survey of facilitators and barriers to recruitment to the MAGNETIC trial. Trials. 2016;17:10.
34. Giatras N, Wanninkhof E, Leontowitsch M, Lewis B, Taylor A, Cooper S, et al. Lessons learned from the London Exercise and Pregnant (LEAP) Smokers randomised controlled trial process

evaluation: implications for the design of physical activity for smoking cessation interventions during pregnancy. Bmc Public Health. 2017;17:11.
35. Girard D, Bourdon O, Abdoul H, Prot-Labarthe S, Brion F, Tibi A, et al. How to improve the implementation of academic clinical pediatric trials involving drug therapy? A qualitative study of multiple stakeholders. PloS one. 2013;8(5):e64516.
36. Harrop E, Kelly J, Griffiths G, Casbard A, Nelson A, TMG BTMG. Why do patients decline surgical trials? Findings from a qualitative interview study embedded in the Cancer Research UK BOLERO trial (Bladder cancer: Open versus Lapararoscopic or RObotic cystectomy). Trials. 2016;17:11.
37. Hetherton J, Matheson A, Robson M. Recruitment by GPs during consultations in a primary care randomized controlled trial comparing computerized psychological therapy with clinical psychology and routine GP care: problems and possible solutions. Primary Health Care Research & Development. 2004;5(1):5-10.
38. Hilliard C, Brenner M. Assimilation into daily practice: implementing and sustaining a randomised-controlled trial in a children's clinical service. Journal of Clinical Nursing. 2016;25(1-2):186-93.
39. Hind D, Parkin J, Whitworth V, Rex S, Young T, Hampson L, et al. Aquatic therapy for children with Duchenne muscular dystrophy: a pilot feasibility randomised controlled trial and mixed-methods process evaluation. Health Technology Assessment. 2017;21(27):I-+.
40. Hinton L, Zweifach M, Oishi S, Tang L, Unutzer J. Gender disparities in the treatment of late-life depression: Qualitative and quantitative findings from the IMPACT trial. American Journal of Geriatric Psychiatry. 2006;14(10):884-92.
41. Horwood J, Johnson E, Gooberman-Hill R. Understanding involvement in surgical orthopaedic randomized controlled trials: A qualitative study of patient and health professional views and experiences. International Journal of Orthopaedic and Trauma Nursing. 2016;20:3-12.
42. Instone SL, Mueller M-R, Gilbert TL. Therapeutic discourse among nurses and physicians in controlled clinical trials. Nursing ethics. 2008;15(6):803-12.
43. J. L. Donovan JAL, T. J. Peters, L. Brindle, E. Salter, D. Gillatt, P. Powell, P. Bollina, D. E. Neal and F. C. Hamdy. Development of a complex intervention improved randomization and informed consent in a randomized controlled trial. Journal of Clinical Epidemiology. 2009;62(1):29-36.
44. J. Raftery JB, J. Powell, C. Kerr and S. Hawker. Payment to healthcare professionals for patient recruitment to trials: systematic review and qualitative study. Health Technology Assessment. 2008;12(10):1-128.
45. Jaspers P, van der Arend A, Wanders R. Inclusion Practice in Lung Cancer Trials. Nursing Ethics. 2006;13(6):649-60.
46. Jepson M, Elliott D, Conefrey C, Wade J, Rooshenas L, Wilson C, et al. An observational study showed that explaining randomization using gambling-related metaphors and computer-agency descriptions impeded randomized clinical trial recruitment. Journal of Clinical Epidemiology. 2018;99:75-83.
47. Kandola DK, Banner D, Araki Y, Bates J, Hadi H, Lear SA. The Participant Recruitment Outcomes (PRO) study: Exploring contemporary perspectives of telehealth trial non-participation through insights from patients, clinicians, study investigators, and study staff. Contemporary clinical trials communications. 2018;11:75-82.
48. Keller PH, Grondin O, Tison F, Gonon F. How Health Professionals Conceptualize and Represent Placebo Treatment in Clinical Trials and How Their Patients Understand It: Impact on Validity of Informed Consent. Plos One. 2016;11(5):17.
49. Lavender V, Gibson F, Brownsdon A, Fern L, Whelan J, Pearce S. Health professional perceptions of communicating with adolescents and young adults about bone cancer clinical trial participation. Supportive Care in Cancer. 2019;27(2):467-75.
50. Lawton J, Hallowell N, Snowdon C, Norman JE, Carruthers K, Denison FC. Written versus verbal consent: a qualitative study of stakeholder views of consent procedures used at the time of

recruitment into a peripartum trial conducted in an emergency setting. BMC Medical Ethics. 2017;18:1-13.
51. Lawton J, Jenkins N, Darbyshire J, Farmer A, Holman R, Hallowell N. Understanding the outcomes of multi-centre clinical trials: A qualitative study of health professional experiences and views. Social Science & Medicine. 2012;74(4):574-81.
52. Lawton J, Jenkins N, Darbyshire JL, Holman RR, Farmer AJ, Hallowell N. Challenges of maintaining research protocol fidelity in a clinical care setting: A qualitative study of the experiences and views of patients and staff participating in a randomized controlled trial. Trials. 2011;12:10.
53. Lawton J, Kirkham J, White D, Rankin D, Cooper C, Heller S. Uncovering the emotional aspects of working on a clinical trial: a qualitative study of the experiences and views of staff involved in a type 1 diabetes trial. Trials. 2015;16:3.
54. Lawton J, Snowdon C, Morrow S, Norman JE, Denison FC, Hallowell N. Recruiting and consenting into a peripartum trial in an emergency setting: a qualitative study of the experiences and views of women and healthcare professionals. Trials. 2016;17:1-14.
55. Lidz CW, Benedicto CM, Albert K, Appelbaum PS, Dunn LB. Clinical Concerns and the Validity of Clinical Trials. AJOB Primary Research. 2013;4(4):26-38.
56. Lie MLS, Lecouturier J, Harding C. Should I stay or should I go? A qualitative study exploring participation in a urology clinical trial. International urogynecology journal. 2019;30(1):9-16.
57. Liu H, Massi L, Eades AM, Howard K, Peiris D, Redfern J, et al. Implementing Kanyini GAP, a pragmatic randomised controlled trial in Australia: findings from a qualitative study. Trials. 2015;16:11.
58. Loh WY, Butow PN, Brown RF, Boyle F. Ethical communication in clinical trials. Issues faced by data managers in obtaining informed consent. Cancer. 2002;95(11):2414-21.
59. Mann C, Delgado D, Horwood J. Evaluation of internal peer-review to train nurses recruiting to a randomized controlled trial - Internal Peer-review for Recruitment Training in Trials (Inter PReTiT). Journal of Advanced Nursing (John Wiley & Sons, Inc). 2014;70(4):777-90.
60. Marley JV, Kitaura T, Atkinson D, Metcalf S, Maguire GP, Gray D. Clinical trials in a remote Aboriginal setting: lessons from the BOABS smoking cessation study. Bmc Public Health. 2014;14:9.
61. Marshman Z, Innes N, Deery C, Hall M, Speed C, Douglas G, et al. The management of dental caries in primary teeth - involving service providers and users in the design of a trial. Trials. 2012;13:9.
62. Maslin-Prothero S. The role of the multidisciplinary team in recruiting to cancer clinical trials. European Journal of Cancer Care. 2006;15(2):146-54.
63. Mason SA, Allmark PJ, Euricon Study G. Obtaining informed consent to neonatal randomised controlled trials: interviews with parents and clinicians in the Euricon study. Lancet. 2000;356(9247):2045-51.
64. Matthias B, Elger Bernice S, Von Elm E, Priya S. Insufficient recruitment and premature discontinuation of clinical trials in Switzerland: qualitative study with trialists and other stakeholders. Swiss Medical Weekly. 2017;147(45-46):w14556.
65. Mills N, Blazeby JM, Hamdy FC, Neal DE, Campbell B, Wilson C, et al. Training recruiters to randomized trials to facilitate recruitment and informed consent by exploring patients' treatment preferences. Trials. 2014;15:13.
66. Mills N, Donovan JL, Wade J, Hamdy FC, Neal DE, Lane JA. Exploring treatment preferences facilitated recruitment to randomized controlled trials. Journal of Clinical Epidemiology. 2011;64(10):1127-36.
67. Mollart L, Adams J, Foureur M. Pregnant women and health professional's perceptions of complementary alternative medicine, and participation in a randomised controlled trial of acupressure for labour onset. Complementary therapies in clinical practice. 2016;24:167-73.
68. Newington L, Metcalfe A. Factors influencing recruitment to research: qualitative study of the experiences and perceptions of research teams. BMC medical research methodology. 2014;14:10.

69. Nichol J, Thompson EA, Shaw A. Health professionals' and families' understanding of the role of individualised homeopathy in asthma management for children requiring secondary care: Qualitative findings from a mixed methods feasibility study. European Journal of Integrative Medicine. 2013;5(5):418-26.
70. Nixon J, Nelson EA, Cranny G, Iglesias CP, Hawkins K, Cullum NA, et al. Pressure relieving support surfaces: A randomised evaluation. Health Technology Assessment. 2006;10(22).
71. Noble SI, Nelson A, Fitzmaurice D, Bekkers M-J, Baillie J, Sivell S, et al. A feasibility study to inform the design of a randomised controlled trial to identify the most clinically effective and cost-effective length of Anticoagulation with Low-molecular-weight heparin In the treatment of Cancer-Associated Thrombosis (ALICAT). Health technology assessment (Winchester, England). 2015;19(83):vii-93.
72. Palmer S, Cramp F, Clark E, Lewis R, Brookes S, Hollingworth W, et al. The feasibility of a randomised controlled trial of physiotherapy for adults with joint hypermobility syndrome. Health Technology Assessment. 2016;20(47):1-290.
73. Paramasivan S, Rogers CA, Welbourn R, Byrne JP, Salter N, Mahon D, et al. Enabling recruitment success in bariatric surgical trials: Pilot phase of the By-Band-Sleeve study. International Journal of Obesity. 2017;41(11):1654-61.
74. Paramasivan S, Strong S, Wilson C, Campbell B, Blazeby JM, Donovan JL. A simple technique to identify key recruitment issues in randomised controlled trials: Q-QAT - quanti-qualitative appointment timing. Trials. 2015;16:15.
75. Pattison N, Arulkumaran N, Humphreys S, Walsh T. Exploring obstacles to critical care trials in the UK: A qualitative investigation. Journal of the Intensive Care Society. 2017;18(1):36-46.
76. Pearce S, Brownsdon A, Fern L, Gibson F, Whelan J, Lavender V. The perceptions of teenagers, young adults and professionals in the participation of bone cancer clinical trials. European journal of cancer care. 2018;27(6):e12476.
77. Peay HL, Scharff H, Tibben A, Wilfond B, Bowie J, Johnson J, et al. "Watching time tick by...": Decision making for Duchenne muscular dystrophy trials. Contemporary Clinical Trials. 2016;46:1-6.
78. Peay HL, Tibben A, Fisher T, Brenna E, Biesecker BB. Expectations and experiences of investigators and parents involved in a clinical trial for Duchenne/Becker muscular dystrophy. Clinical trials (London, England). 2014;11(1):77-85.
79. Penn C, Frankel T, Watermeyer J, Muller M. Informed consent and aphasia: evidence of pitfalls in the process. Aphasiology. 2009;23(1):3-32.
80. Prout H, Butler C, Kinnersley P, Robling M, Hood K, Tudor-Jones R. A qualitative evaluation of implementing a randomized controlled trial in general practice. Family Practice. 2003;20(6):675-81.
81. Quirk H, Glazebrook C, Blake H. A physical activity intervention for children with type 1 diabetes-steps to active kids with diabetes (STAK-D): a feasibility study. Bmc Pediatrics. 2018;18:12.
82. Raftery J, Kerr C, Hawker S, Powell J. Paying clinicians to join clinical trials: a review of guidelines and interview study of trialists. Trials. 2009;10:15.
83. Rendon JS, Swinton M, Bernthal N, Boffano M, Damron T, Evaniew N, et al. Barriers and facilitators experienced in collaborative prospective research in orthopaedic oncology. Bone Jt Res. 2017;6(5):307-14.
84. Rooshenas L, Elliott D, Wade J, Jepson M, Paramasivan S, Strong S, et al. Conveying Equipoise during Recruitment for Clinical Trials: Qualitative Synthesis of Clinicians' Practices across Six Randomised Controlled Trials. Plos Medicine. 2016;13(10):24.
85. S. K. McCann MKCaVAE. Reasons for participating in randomised controlled trials: conditional altruism and considerations for self. Trials. 2010;11.
86. Sayal K, Roe J, Ball H, Atha C, Kaylor-Hughes C, Guo BL, et al. Feasibility of a randomised controlled trial of remotely delivered problem-solving cognitive behaviour therapy versus usual care for young people with depression and repeat self-harm: lessons learnt (e-DASH). Bmc Psychiatry. 2019;19:12.

87. Shepherd V, Thomas-Jones E, Ridd MJ, Hood K, Addison K, Francis NA. Impact of a deferred recruitment model in a randomised controlled trial in primary care (CREAM study). Trials. 2017;18(1):533.
88. Shilling V, Williamson PR, Hickey H, Sowden E, Beresford MW, Smyth RL, et al. Communication about children's clinical trials as observed and experienced: qualitative study of parents and practitioners. PloS one. 2011;6(7):e21604.
89. Shilling V, Williamson PR, Hickey H, Sowden E, Smyth RL, Young B. Processes in recruitment to randomised controlled trials of medicines for children (RECRUIT): A qualitative study. Health Technology Assessment. 2011;15(15):I-116.
90. Sifunda S, Reddy P, Naidoo N, James S, Buchanan D. Recruiting and Educating Participants for Enrollment in HIV-Vaccine Research: Ethical Implications of the Results of an Empirical Investigation. Public Health Ethics. 2014;7(1):78-85.
91. Spilsbury K, Petherick E, Cullum N, Nelson A, Nixon J, Mason S. The role and potential contribution of clinical research nurses to clinical trials. Journal of Clinical Nursing (Wiley-Blackwell). 2008;17(4):549-57.
92. van der Zande ISE, van der Graaf R, Oudijk MA, van Vliet-Lachotzki EH, van Delden JJM. A qualitative study on stakeholders' views on the participation of pregnant women in the APOSTEL VI study: a low-risk obstetrical RCT. Bmc Pregnancy and Childbirth. 2019;19:11.
93. van Staa T-P, Dyson L, McCann G, Padmanabhan S, Belatri R, Goldacre B, et al. The opportunities and challenges of pragmatic point-of-care randomised trials using routinely collected electronic records: evaluations of two exemplar trials. Health Technology Assessment. 2014;18(8):1-146.
94. Vemulakonda VM, Jones J. Barriers to participation in surgical randomized controlled trials in pediatric urology: A qualitative study of key stakeholder perspectives. J Pediatr Urol. 2016;12(3):7.
95. Wade J, Donovan JL, Athene Lane J, Neal DE, Hamdy FC. It's not just what you say, it's also how you say it: Opening the 'black box' of informed consent appointments in randomised controlled trials. Social Science and Medicine. 2009;68(11):2018-28.
96. Watson DLB, Sanoff R, Mackintosh JE, Saver JL, Ford GA, Price C, et al. Evidence From the Scene: Paramedic Perspectives on Involvement in Out-of-Hospital Research. Annals of Emergency Medicine. 2012;60(5):641-50.
97. Whybrow P, Pickard R, Hrisos S, Rapley T. Equipoise across the patient population: optimising recruitment to a randomised controlled trial. Trials. 2017;18:12.
98. Woolfall K, Frith L, Gamble C, Gilbert R, Mok Q, Young B, et al. How parents and practitioners experience research without prior consent (deferred consent) for emergency research involving children with life threatening conditions: a mixed method study. Bmj Open. 2015;5(9):14.
