# Supplementary material for: Understanding the perspectives of recruiters is key to improving randomised controlled trial enrolment: a qualitative evidence synthesis
Source: Trials. 2022 Oct 20;23:883. doi: 10.1186/s13063-022-06818-4 (PMC9585862; doi:10.1186/s13063-022-06818-4)
Supplement: Supplementary file 4 — Additional file 4. CASP. Critical Appraisal Skills Programme for appraising a qualitative study. [file 13063_2022_6818_MOESM4_ESM.pdf]

Understanding the perspectives of recruiters is key to improving randomised controlled trial enrolment: a qualitative evidence synthesis – Additional File 4

	Assessor	*Was there a clear statement of the aims of the research?*	*Is a qualitative methodology appropriate?*	*Was the research design appropriate to address the aims of the research?*	*Was the recruitment strategy appropriate to the aims of the research?*	*Was the data collected in a way that addressed the research issue?*	*Has the relationship between researcher and participants been adequately considered?*	*Have ethical issues been taken into consideration?*	*Was the data analysis sufficiently rigorous?*	*Is there a clear statement of findings?*	*How valuable is the research? (Is the research valuable)*	*Overall assessment*
**Campbell, M., et al**	NF	Yes	Yes	Yes	Yes	Yes	Yes	Yes	Yes	Yes	Yes	No or very minor concerns
	Reviewer 2	Yes	Yes	Yes	Yes	Yes	Yes	Yes	Yes	Yes	Yes	
**Clement, C., et al**	NF	Yes	Yes	Yes?	Yes?	Yes	Unclear	Yes	Yes	Yes	Yes	No or very minor concerns
	Reviewer 2	Yes	Yes	Yes	Yes	Yes	Yes?	Yes	Yes?	Yes	Yes	
**Donovan, J., et al**	NF	Yes	Yes	Yes	Yes	Yes	Unclear	Yes	Yes	Yes	Yes	No or very minor concerns
	Reviewer 2	Yes	Yes	Yes	Yes	Yes	Unclear	Yes	Yes	Yes	Yes	
**Donovan, J., et al**	NF	Yes	Yes	Yes	Yes	Yes	No	Unclear	Yes	Yes	Yes	No or very minor concerns
	Reviewer 2	Yes	Yes	Yes	Yes	Yes	No	Yes	Yes	Yes	Yes	
**Ekambareshwar, M., et al**	NF	Yes	Yes	Yes?	Unclear	Yes?	No	Yes	Yes?	Yes	Yes	Minor concerns
	Reviewer 2	Yes	Yes	Yes?	Unclear	Yes?	No	Yes	Yes?	Yes	Yes	
**Frayne, S., et al**	NF	Yes	Yes	Yes	Yes	Yes	No	Yes	Yes?	Yes	Yes	Minor concerns
	Reviewer 2	Yes	Yes	Yes	Yes	Yes	No	Yes	Yes?	Yes	Yes	
**Griffin, D., et al**	NF	Yes	Yes	Yes	Yes	Yes	Unclear	Yes?	Yes	Yes	Yes	No or very minor concerns
	Reviewer 2	Yes	Yes	Yes	Yes	Yes	Unclear	yes?	Yes	Yes	Yes	

**Hallowell, N., et al**	NF	Yes	Yes	Yes	Yes	Yes	No	Yes	Yes	Yes	Yes	No or very minor concerns
	Reviewer 2	Yes	Yes	Yes	Yes	Yes	No	Yes	Yes	Yes	Yes	
**Hamdy, F., et al**	NF	Yes	Yes	Yes	Yes	Yes	Unclear	Yes	Yes	Yes	Yes	No or very minor concerns
	Reviewer 2	Yes	Yes	Yes	Yes	Yes	No	Yes	Yes	Yes	Yes	
**Hamilton, D., et al**	NF	Yes	Yes	Yes	Yes?	Yes	No	Yes	Yes?	Yes	Yes	No or very minor concerns
	Reviewer 2	Yes	Yes	Yes	Yes?	Yes	No	Yes	Yes?	Yes	Yes	
**Hamlet, C., et al**	NF	Yes	Yes	Yes	Yes?	Yes	Yes?	Unclear	Yes?	Yes	Yes	No or very minor concerns
	Reviewer 2	Yes	Yes	Yes	Yes	Yes	Yes	Yes	Yes	Yes	yes	
**Hange, D., et al**	NF	Yes	Yes	Yes	Yes	Yes	Yes	Yes	Yes?	Yes	Yes	Minor concerns
	Reviewer 2	Yes	Yes	Yes	Yes	Yes	Yes	Yes	Yes	Yes	Yes	
**Hanson, L., et al**	NF	Yes	Yes	Yes	Yes	Yes	Unclear	Yes	Yes?	Yes	Yes	No or very minor concerns
	Reviewer 2	Yes	Yes	Yes	Yes	Yes	Unclear	Yes	Yes?	Yes	Yes	
**Holm, M., et al**	NF	Yes	Yes	Yes	Yes	Yes	Unclear	Yes	Yes	Yes	Yes	No or very minor concerns
	Reviewer 2	Yes	Yes	Yes	Yes	Yes	Yes	Yes	Yes	Yes	Yes	
**Howard, L., et al**	NF	Yes	Yes	Yes	Yes?	Yes?	No	No	Yes?	Yes	Yes	Minor concerns
	Reviewer 2	Yes	Yes	Yes	Yes?	Yes?	No	No	Yes?	Yes	Yes	
**Langley, C., et al**	NF	Yes	Yes	Yes	Yes	Yes	No	Unclear	Yes	Yes	Yes	No or very minor concerns
	Reviewer 2	Yes	Yes	Yes	Yes	Yes	No	Unclear	Yes	Yes	Yes	

**Mason, V., et al**	NF	Yes	Yes	Yes	Yes	Yes	Yes	No	Yes	Yes	Yes	No or very minor concerns
	Reviewer 2	Yes	Yes	Yes	Yes	Yes	Yes	No	Yes	Yes	Yes	
**Paleri, V., et al**	NF	Yes	Yes	Yes	Yes	Yes	Yes?	Yes	Yes	Yes	Yes	No or very minor concerns
	Reviewer 2	Yes	Yes	Yes	Yes	Yes	Yes?	Yes	Yes	Yes	Yes	
**Paramasivan, S., et al**	NF	Yes	Yes	Yes	Yes	Yes	Unclear	Yes	Yes	Yes	Yes	No or very minor concerns
	Reviewer 2	Yes	Yes	Yes	Yes	Yes	Unclear	Yes	Yes	Yes	Yes	
**Phelps, E., et al**	NF	Yes	Yes	Yes	Yes	Yes	Yes	Yes	Yes	Yes	Yes	No or very minor concerns
	Reviewer 2	Yes	Yes	Yes	Yes	Yes	Yes	Yes	Yes	Yes	Yes	
**Potter, R., et al**	NF	Yes	Yes	Yes	Yes	Yes	Unclear	Yes	Yes	Yes	Yes	No or very minor concerns
	Reviewer 2	Yes	Yes	Yes	Yes	Yes?	Unclear	Yes	Yes	Yes	Yes	
**Shaheed, C., et al**	NF	Yes	Yes	Yes?	Yes?	Yes?	No	Yes?	Unclear	Yes	Unclear	Moderate concerns
	Reviewer 2	Yes	Yes	Yes?	Yes?	Yes?	No	Yes?	Unclear	Yes	Unclear	
**Sin, J., et al**	NF	Yes	Yes	Yes	Yes	Yes	No	Yes	Yes	Yes	Yes	No or very minor concerns
	Reviewer 2	Yes	Yes	Yes	Yes	Yes	No	Yes	Yes	Yes	Yes	
**Skea, Z., et al**	NF	Yes	Yes	Yes	Yes	Yes	Yes	Yes	Yes	Yes	Yes	No or very minor concerns
	Reviewer 2	Yes	Yes	Yes	Yes	Yes	Yes	Yes	Yes	Yes	Yes	
**Stein, R., et al**	NF	Yes	Yes	Yes	Yes	Yes	Yes	Yes	Yes	Yes	Yes	No or very minor concerns
	Reviewer 2	Yes	Yes	Yes	Yes	Yes	Yes	Yes	Yes	Yes	Yes	

**Strong, S., et al**	NF	Yes	Yes	Yes	Yes	Yes	Yes	Yes	Yes	Yes	Yes	No or very minor concerns
	Reviewer 2	Yes	Yes	Yes	Yes	Yes	Yes	Yes	Yes	Yes	Yes	
**Team, V., et al**	NF	Yes	Yes	Yes	Yes?	Yes	No	Yes	Yes	Yes	Yes	No or very minor concerns
	Reviewer 2	Yes	Yes	Yes	Yes?	Yes	No	Yes	Yes	Yes	Yes	
**Tomlin, Z., et al**	NF	Yes	Yes	Yes	Yes?	Yes	Yes	Yes	Yes	Yes	Yes	No or very minor concerns
	Reviewer 2	Yes	Yes	Yes	Yes?	Yes	Yes	Yes	Yes	Yes	Yes	
**Wright, J., et al**	NF	Yes	Yes	Yes	Yes	Yes	Unclear	Unclear	Unclear	Yes	Yes	Minor concerns
	Reviewer 2	Yes	Yes	Yes	Yes	Yes	Unclear	Unclear	Unclear	Yes	Yes	
**Ziebland, S., et al**	NF	Yes	Yes	Yes	Yes	Yes?	Yes?	Yes	Yes	Yes	Yes	No or very minor concerns
	Reviewer 2	Yes	Yes	Yes	Yes	Yes?	Yes?	Yes	Yes	Yes	Yes	
