# Supplementary material for: Understanding the perspectives of recruiters is key to improving randomised controlled trial enrolment: a qualitative evidence synthesis
Source: Trials. 2022 Oct 20;23:883. doi: 10.1186/s13063-022-06818-4 (PMC9585862; doi:10.1186/s13063-022-06818-4)
Supplement: Supplementary file 6 — Additional file 6. Full EP. GRADE CERQual full evidence profile. [file 13063_2022_6818_MOESM6_ESM.pdf]

Understanding the perspectives of recruiters is key to improving randomised controlled trial enrolment: a qualitative evidence synthesis – Additional File 6

Summary of review finding	Studies contributing to the review finding	Methodological limitations	Coherence	Adequacy	Relevance	CERQual assessment	Explanation of CERQual assessment
Theme 1: Recruiting to RCTs in a clinical environment							
1. Recruiters generally expressed that identifying eligible patients for an RCT was difficult to do in normal clinical practice, recognising factors such as the way patients engaged with clinical services, insufficient record keeping, and there being fewer eligible patients than anticipated as impacting the opportunity to identify patients	(1-14)<br><br>14	**Minor concern** based on the assessment of 12 studies of no/very minor concern, 1 of minor concern and 1 of moderate concern	No or very minor concerns	No or very minor concerns	**Minor concern** based on the assessment of 9 studies of whole relevance and 5 studies of partial relevance	High confidence	No or very minor concerns about methodological limitations and coherence, minor concerns about adequacy and relevance
2. Recruiters identified that there were issues related to patients' abilities or motivations to attend visits related to the	(1, 4, 8, 14-18)<br><br>8	**Minor concern** based on the assessment of 6 studies of no/very minor concern, 1 of	No or very minor concerns	Minor concerns	**Minor concern** based on the assessment of 6 studies of	High confidence	No or very minor concerns about coherence, minor concerns about

	RCT, which impacted recruitment. These included practical issues such as accessing hospitals and committing time to the RCT		minor concern and 1 of moderate concern			whole relevance and 2 of partial relevance		methodological limitations, adequacy and relevance
3.	Recruiters felt that they had insufficient time to dedicate to recruitment as they had other commitments and busy workloads. This was particularly prevalent for primary care recruiters	(1, 3, 5, 6, 8, 9, 14, 18-21)<br><br>11	**Minor concern** based on the assessment of 9 studies of no/very minor concerns, 1 of minor concerns, 1 of minor concern and 1 of moderate concern	No or very minor concerns	No or very minor concerns	**Minor concern** based on the assessment of 9 studies of whole relevance and 2 of partial relevance	High confidence	No or very minor concerns about coherence and adequacy, minor concerns about methodological limitations and relevance
4.	Recruiters generally noted the value of sufficient staff resource, and the benefit of having additional recruitment support was recognised. Recruiters perceived that if they had additional resources (such as nurse time) it would	(2, 3, 7, 12, 14, 19, 20, 22)<br><br>8	**No or very minor concerns**	Minor concerns	No or very minor concerns	**No or very minor concerns** based on the assessment of 9 studies of whole relevance and	High confidence	No or very minor concerns about methodological limitations, adequacy and relevance, minor

	be beneficial for recruitment					1 of partial relevance		concerns about coherence
5.	Recruiters sometimes had attitudes towards the RCTs that were understood to not be conducive to recruitment, such as suspicion towards research and believing there were other priorities over recruiting patients. These attitudes were often rooted in a belief that day to day care of patients should take precedence	(1-3, 5, 6, 10, 13, 20-24)	**Minor concerns** based on the assessment of 9 studies of no/very minor concern, 2 of minor concern, and 1 of moderate concern	Moderate concerns	Minor concerns	**Minor concerns** based on the assessment of 8 studies of whole relevance and 4 of partial relevance	Moderate confidence	Minor concern about methodological limitations, adequacy and relevance, moderate concern about coherence
6.	A culture of research at a site was believed to be beneficial to recruitment, as in some cases it encouraged engagement from the wider clinical team. What constituted a research culture was not uniformly defined	(3, 12, 21, 25)	**No or very minor concerns**	No or very minor concerns	Moderate concerns	**No or very minor concerns**	Moderate confidence	No or very minor concerns about methodological limitations, coherence and relevance,

and varied between RCTs							moderate concern about adequacy
Theme 2: Recruiters' enthusiasm for the RCT							
7. Enthusiasm for an RCT was considered beneficial for recruitment and often stemmed from a desire to improve patient care and outcomes, and resolve uncertainty	(2, 4-6, 10, 14-17, 19-21, 23, 26-28)	**No or very minor concerns** based on the assessment of 14 studies with no or very minor concerns and 2 of minor concern	No or very minor concerns	No or very minor concerns	**Minor concern** based on the assessment of 10 studies of whole relevance and 6 of partial relevance	High confidence	No or very minor concern about methodological limitations, coherence or adequacy, minor concerns about relevance
8. Some recruiters identified that motivation to recruit participants came from a desire to access treatments available within the RCT as they believed them to be beneficial	(2, 18)	**No or very minor concerns**	Moderate concern	Serious concerns based on both the volume and thickness of data	**Minor concern** based on the assessment of 1 study of whole relevance and	Low confidence	No or very minor concern about methodological limitations, minor concern about relevance, moderate concern about coherence

					1 of partial relevance		and serious concerns about adequacy
9. Obtaining support from team members who were not directly associated with recruitment was considered to be important, as a supportive clinical team was understood to improve the recruitment process, but this could be difficult to achieve and could require ongoing effort on the part of the recruiters	(2, 7, 8, 10-12, 22, 25, 26, 29)	**No or very minor concerns** based on the assessment of 9 studies with no or very minor concerns and 1 study of minor concern	No or very minor concerns	No or very minor concerns	**Minor concern** based on the assessment of 7 studies of whole relevance and 3 of partial relevance	High confidence	No or very minor concern about methodological limitations, coherence and adequacy, minor concern about relevance
Theme 3: Making judgements about whether to approach a patient							
10. Not all patients who are eligible to take part in the RCT are approached, with recruiters applying inclusion and exclusion criteria variably and	(4, 5, 9, 11, 13, 17, 19, 21, 23, 25, 28, 30)	**No or very minor concerns** based of 11 studies of no or very	No or very minor concerns	No or very minor concerns	**No or very minor concerns** based on the assessment of	High confidence	No or very minor concern about methodological limitations, coherence

suggesting reasons outside of these criteria why patients may not be approached, such as having preferences for patients to receive certain treatments		minor concern and 1 of minor concern			10 studies of whole relevance and 2 of partial relevance		adequacy, and relevance
11. Discomfort about recruiting patients due to the appropriateness of the eligibility criteria can be pronounced when recruiters believe that patients are on the periphery of the eligibility criteria. The prevalence of this struggle was highlighted in particular for recruiters who were also clinicians when they felt less (un)certain about the best treatment for a patient	(4, 10, 13, 14, 17, 19, 23, 25)	**No or very minor concerns**	Moderate concern	Minor concerns	**No or very minor concerns** based on the assessment of 7 studies of whole relevance and 1 of partial relevance	Moderate confidence	No or very minor concerns about methodological limitations and relevance, minor concern about adequacy and moderate concern about coherence

12. Recruiters often made additional judgements about individual patients' personal, social or other non-clinical factors when deciding whether to approach them about RCT participation. This sometimes resulted in recruiters acting as 'gatekeepers' and denying patients the opportunity to make a decision for themselves	(2, 4, 5, 9-11, 13, 14, 16, 18-21, 25, 27-30)	**No or very minor concerns** based on the assessment of 16 studies of no/very minor concern and 2 of minor concern	No or very minor concerns	No or very minor concerns	Minor concerns based on the assessment of 13 studies of whole relevance and 5 of partial relevance	High confidence	No or very minor concerns about methodological limitations, coherence and adequacy, minor concern about relevance
13. Recruiters also reported that others involved in the patient's care may act as gatekeepers, preventing access to their patients who may be suitable for inclusion in the RCT	(3, 7, 9, 22, 29)	**Minor concern** based on assessment of 4 studies of no/very minor concern and 1 of minor concern	No or very minor concerns	Moderate concerns	**No or very minor concerns**	Moderate confidence	No or very minor concern about coherence or relevance, minor concern about methodological limitations and moderate concern about adequacy

Theme 4: Communication challenges							
14. Some recruiters, who approached patients despite having treatment preferences, were found to influence patient preferences when doing so, such as by giving direct treatment recommendations. They also believed that other members of staff had preferences for certain treatments, which also influenced patients' preferences	(4, 6, 10, 11, 13, 17, 19, 22, 23)	**No or very minor concerns** based on the assessment of 8 studies of no/very minor concerns and 1 of minor concern	Minor concern	Minor concerns	Minor concerns based on the assessment of 6 studies of whole relevance and 3 of partial relevance	Moderate confidence	No or very minor concerns about methodological limitations, minor concerns about coherence, adequacy and relevance
15. Difficulties explaining the study to patients were noted and terms which were perceived by study authors to be 'problematic' were often used to describe elements of the RCT, such as randomisation. The use of problematic terms was understood	(3, 6, 8, 11-13, 16, 17, 19-21, 28, 30)	**No or very minor concerns** based on the assessment of 11 studies of no/very minor concerns and 2 of minor concern	Minor concern	No or very minor concerns	**Minor concerns** based on the assessment of 10 studies of whole relevance and	High confidence	No or very minor concerns about methodological limitations and adequacy, minor concern about

to act as a barrier to recruitment					3 of partial relevance		coherence and relevance
16. Recruiters believed that patients often had preferences for particular treatments. Recruiters often accepted patients' treatment preferences, with justifications for doing so being influenced by recruiters' personal beliefs	(1, 4, 10-13, 17, 19, 20)	**Minor concern** based on the assessment of 8 studies of no/very minor concerns and 1 of moderate concern	No or very minor concerns	No or very minor concerns	**Minor concern** based on the assessment of 6 studies of whole relevance and 3 of partial relevance	High confidence	No or very minor concern about coherence or adequacy, minor concern about methodological limitations and relevance
Theme 5: Interplay between recruiter and professional roles							
17. Recruiters with clinical or other roles in addition to their recruitment roles had to strike a balance between the two roles, often acting both as a recruiter and an advocate for the patient. This was particularly prevalent for nurse recruiters	(2, 4, 5, 8, 10, 13, 14, 18, 21, 23-25, 27, 30)	**No or very minor concerns** based on assessment of 12 studies of no/very minor concerns and 2 of minor concerns	No or very minor concerns	No or very minor concerns	**Minor concern** based on the assessment of 11 studies of whole relevance and	High confidence	No or very minor concerns about methodological limitations, coherence or adequacy, minor

who reported conflict between the roles					3 of partial relevance		concern about relevance
18. Whilst the 'dual role' of being a clinician and recruiter was acknowledged, the clinical role was often prioritised. Traditional perceptions of recruiters' own roles emphasised their caring and advocacy responsibilities, which took precedence over their recruitment role and could not be encroached upon. This was identified in particular for nurses and GPs	(2, 4-6, 21, 24, 25, 27)	**Minor concern** based on the assessment of 6 studies of no/very minor concerns and 2 of minor concern	Minor concern	Minor concern	**Minor concern** based on the assessment of 5 studies of whole relevance and 3 of partial relevance	Moderate confidence	Minor concern about methodological limitations, coherence, adequacy and relevance

1. Shaheed CA, Maher CG, Williams KA, McLachlan AJ. Participation of pharmacists in clinical trial recruitment for low back pain. International Journal of Clinical Pharmacy. 2014;36(5):986-94.
2. Campbell MK, Snowdon C, Francis D, Elbourne D, McDonald AM, Knight R, et al. Recruitment to randomised trials: strategies for trial enrolment and participation study. The STEPS study. Health Technol Asses. 2007;11(48):113.

3. Clement C, Selman LE, Kehoe PG, Howden B, Lane JA, Horwood J. Challenges to and facilitators of recruitment to an Alzheimer's disease clinical trial: A qualitative interview study. Journal of Alzheimer's Disease. 2019;69(4):1067-75.
4. Donovan JL, Paramasivan S, de Salis I, Toerien M. Clear obstacles and hidden challenges: understanding recruiter perspectives in six pragmatic randomised controlled trials. Trials. 2014;15.
5. Hamlet C, Williamson H, Harcourt D. Recruiting young people with a visible difference to the YP Face IT feasibility trial: A qualitative exploration of primary care staff experiences. Primary Health Care Research and Development. 2017;18(6):541-8.
6. Hange D, Bjorkelund C, Svenningsson I, Kivi M, Eriksson MC, Petersson EL. Experiences of staff members participating in primary care research activities: A qualitative study. International Journal of General Medicine. 2015;8:143-8.
7. Hanson LC, Bull J, Wessell K, Massie L, Bennett RE, Kutner JS, et al. Strategies to Support Recruitment of Patients With Life-Limiting Illness for Research: The Palliative Care Research Cooperative Group. J Pain Symptom Manage. 2014;48(6):1021-30.
8. Holm M, Alvariza A, Fürst C-J, Wengström Y, Årestedt K, Öhlen J, et al. Recruiting participants to a randomized controlled trial testing an intervention in palliative cancer care – The perspectives of health care professionals. European Journal of Oncology Nursing. 2017;31:6-11.
9. Sin J, Henderson C, Spain D, Gamble C, Norman I. What factors influence successful recruitment of siblings of individuals with first episode psychosis to e-health interventions? A qualitative study. Health Expectations. 2016.
10. Paleri V, Patterson J, Rousseau N, Moloney E, Craig D, Tzelis D, et al. Gastrostomy versus nasogastric tube feeding for chemoradiation patients with head and neck cancer: The tube pilot RCT. Health Technol Asses. 2018;22(16).
11. Paramasivan S, Huddart R, Hall E, Lewis R, Birtle A, Donovan JL. Key issues in recruitment to randomised controlled trials with very different interventions: a qualitative investigation of recruitment to the SPARE trial (CRUK/07/011). Trials. 2011;12:15.
12. Skea ZC, Treweek S, Gillies K. It's trying to manage the work': A qualitative evaluation of recruitment processes within a UK multicentre trial. Bmj Open. 2017;7(8):e016475.
13. Stein RC, Dunn JA, Bartlett JMS, Campbell AF, Marshall A, Hall P, et al. OPTIMA prelim: a randomised feasibility study of personalised care in the treatment of women with early breast cancer. Health technology assessment (Winchester, England). 2016;20(10):xxiii-201.
14. Team V, Bugeja L, Weller CD. Barriers and facilitators to participant recruitment to randomised controlled trials: A qualitative perspective. International Wound Journal. 2018;15(6):929-42.
15. Ekambareshwar M, Mihrshahi S, Wen LM, Taki S, Bennett G, Baur LA, et al. Facilitators and challenges in recruiting pregnant women to an infant obesity prevention programme delivered via telephone calls or text messages. Trials. 2018;19:9.
16. Griffin D, Wall P, Realpe A, Adams A, Parsons N, Hobson R, et al. UK FASHIoN: feasibility study of a randomised controlled trial of arthroscopic surgery for hip impingement compared with best conservative care. Health technology assessment (Winchester, England). 2016;20(32):1-172.
17. Hamilton DW, De Salis I, Donovan JL, Birchall M. The recruitment of patients to trials in head and neck cancer: A qualitative study of the EaStER trial of treatments for early laryngeal cancer. European Archives of Oto-Rhino-Laryngology. 2013;270(8):2333-7.
18. Potter R, Dale J, Caramlau I. A qualitative study exploring practice nurses' experience of participating in a primary care-based randomised controlled trial. Journal of Research in Nursing. 2009;14(5):439-47.

19. Hamdy FC, Elliott D, Le Conte S, Davies LC, Burns RM, Thomson C, et al. Partial ablation versus radical prostatectomy in intermediate-risk prostate cancer: The PART feasibility RCT. Health Technol Asses. 2018;22(52):1-95.
20. Langley C, Gray S, Selley S, Bowie C, Price C. Clinicians' attitudes to recruitment to randomised trials in cancer care: a qualitative study. Journal of health services research & policy. 2000;5(3):164-9.
21. Mason VL, Shaw A, Wiles NJ, Mulligan J, Peters TJ, Sharp D, et al. GPs' experiences of primary care mental health research: a qualitative study of the barriers to recruitment. Fam Pract. 2007;24(5):518-25.
22. Strong S, Paramasivan S, Mills N, Wilson C, Donovan JL, Blazeby JM. 'The trial is owned by the team, not by an individual': a qualitative study exploring the role of teamwork in recruitment to randomised controlled trials in surgical oncology. Trials. 2016;17:1-9.
23. Donovan JL, de Salis I, Toerien M, Paramasivan S, Hamdy FC, Blazeby JM. The intellectual challenges and emotional consequences of equipoise contributed to the fragility of recruitment in six randomized controlled trials. J Clin Epidemiol. 2014;67(8):912-20.
24. Frayne SM, Mancuso M, Prout MN, Freund KM. Attitudes of primary care physicians toward cancer-prevention trials: A focus group analysis. Journal of the National Medical Association. 2001;93(11):450-7.
25. Phelps EE, Tutton E, Griffin X, Baird J, Costa ML, Parsons N, et al. Facilitating trial recruitment: A qualitative study of patient and staff experiences of an orthopaedic trauma trial. Trials. 2019;20(1):492.
26. Hallowell N, Snowdon C, Morrow S, Norman JE, Denison FC, Lawton J. The role of therapeutic optimism in recruitment to a clinical trial in a peripartum setting: balancing hope and uncertainty. Trials. 2016;17:13.
27. Tomlin Z, deSalis I, Toerien M, Donovan JL. Patient advocacy and patient centredness in participant recruitment to randomized-controlled trials: implications for informed consent. Health Expectations. 2014;17(5):670-82.
28. Ziebland S, Featherstone K, Snowdon C, Barker K, Frost H, Fairbank J. Does it matter if clinicians recruiting for a trial don't understand what the trial is really about? Qualitative study of surgeons' experiences of participation in a pragmatic multi-centre RCT. Trials. 2007;8:4.
29. Wright JR, Crooks D, Ellis PM, Mings D, Whelan TJ. Factors that influence the recruitment of patients to phase III studies in oncology - The perspective of the clinical research associate. Cancer. 2002;95(7):1584-91.
30. Howard L, de Salis I, Tomlin Z, Thornicroft G, Donovan J. Why is recruitment to trials difficult? An investigation into recruitment difficulties in an RCT of supported employment in patients with severe mental illness. Contemp Clin Trials. 2009;30(1):40-6.
