# Supplementary material for: Understanding the perspectives of recruiters is key to improving randomised controlled trial enrolment: a qualitative evidence synthesis
Source: Trials. 2022 Oct 20;23:883. doi: 10.1186/s13063-022-06818-4 (PMC9585862; doi:10.1186/s13063-022-06818-4)
Supplement: Supplementary file 7 — Additional file 7. Study Characteristics. Characteristics of each of the studies included in the synthesis. [file 13063_2022_6818_MOESM7_ESM.pdf]

Understanding the perspectives of recruiters is key to improving randomised controlled trial enrolment: a qualitative evidence synthesis – Additional File 7

Study author	Year	Title	Country	Aim	Healthcare setting	Number of RCT(s) and clinical context	Data collection method	Recruiters	Timing of qualitative research
Campbell, M., et al (1)	2007	Recruitment to randomised trials: strategies for trial enrolment and participation study. The STEPS study	UK	Interviewees with a range of roles (e.g. nurse, GP) within an RCT were interviewed with the intention of exploring "the unique circumstances potentially relevant to recruitment for each trial. This element of the research aims to understand, through the opinions of the interviewees, how recruitment may be affected in a very broad sense, and so focuses on their progress, any challenges that arose and any adaptations that were made." [p.20]	Mix of primary and secondary	4 :<br>FOCUS (Cancer)<br>TOuCAN (Adolescent psychiatry)<br>HPS (Cardiovascular, health promotion)<br>ELEVATE (Asthma management)	Interviews	**16**<br>1 Principal Investigator (PI)<br>2 Central recruiters<br>2 Local lead investigators<br>2 Recruiting doctors<br>9 Recruiting nurses	Post RCT recruitment period
Clement, C., et al (2)	2019	Challenges to and Facilitators of Recruitment to an Alzheimer's Disease Clinical Trial: A Qualitative Interview Study	UK	A qualitative study was undertaken during recruitment to the RADAR RCT which "aimed to identify potential challenges to, and facilitators of, recruitment to the RADAR RCT and to draw out implications for improving recruitment to Alzheimer's disease trials more widely" [p.1068]	Secondary	1:<br>RADAR (Alzheimer's disease)	Interviews	**17**<br>7 Doctors<br>10 Nurses	During RCT recruitment period
Donovan, J., et al (3)	2014	Clear obstacles and hidden challenges: understanding recruiter perspectives in six pragmatic randomised controlled trials	UK	"This study aimed i) to understand the recruitment process from the perspective of recruiters actively recruiting RCT participants in six pragmatic RCTs, and ii) to identify opportunities for interventions to improve recruitment." [p.1]	Mix of primary and secondary	6 - not named but described (Cancer x3, primary care and cancer, primary care and infection, mental health and community care)	Interviews	**86**<br>32 Doctors, Chief Investigators (CIs) or Trial Management Group (TMG)<br>40 Nurses<br>15 follow up	During RCT recruitment period
Donovan, J., et al (4)	2014	The intellectual challenges and emotional consequences of equipoise contributed to	UK	"The aim of the study was to investigate how doctors considered and experienced the concept of equipoise while recruiting patients	Mix of primary and secondary	6 - EaStER (Surgery and oncology)<br>FACS (Primary care and oncology)	Interviews	**47**<br>21 Surgeons<br>3 GPs	During RCT recruitment period

		the fragility of recruitment in six randomized controlled trials		to randomized controlled trials" [p.912]		PITCH (Primary care and paediatrics) SWAN (Psychiatry, community services) ProtecT (Surgery and oncology) SPARE (Oncology and surgery)		3 Community mental health 5 Oncologists 15 Follow up interviews	
Ekambareshwar, M., et al (5)	2018	Facilitators and challenges in recruiting pregnant women to an infant obesity prevention programme delivered via telephone calls or text messages	Australia	"This paper focused on the trial recruitment process of the CHAT RCT, to identify facilitators and challenges in recruiting pregnant women to a low intensity health promotion programme where health behaviour change messages are delivered via telephone calls or text messages." [p.2]	Secondary	1 - CHAT RCT (Health promotion in pregnancy)	Interviews	**4** 4 Recruiters	During recruitment period
Frayne, S., et al (6)	2001	Attitudes of primary care physicians toward cancer-prevention trials: a focus group analysis	USA	"We sought to characterize primary care physicians' perceptions of patient-level and physician level barriers to subject recruitment for cancer-prevention trials." [p.451]	Primary	1-BCPT trial (Breast cancer)	Focus group	**7** 7 Physicians	During recruitment period
Griffin, D., et al (7)	2016	UK FASHIoN: feasibility study of a randomised controlled trial of arthroscopic surgery for hip impingement compared with best conservative care	UK	Within the context of the UK FASHIoN RCT, qualitative research methods were used to "1) understand the recruitment process so that any difficulties related to design or conduct can be identified and changes put in place. 2) determine any staff training needs and develop a strategy to address these needs." This work was undertaken with the hope of optimising recruitment to a future full RCT [p.43]	Secondary	1 - UK FASHIoN (Hip impingement)	Interviews and audio-recorded consultations	**21 + 87** **Interviews**: 21 Clinicians and research associates **Audio-recorded consultations:** 87 diagnostic and recruitment consultations	During recruitment period
Hallowell, N., et al (8)	2016	The role of therapeutic optimism in recruitment to a clinical trial in a peripartum setting:	UK	"The aim of the qualitative research was to evaluate staffs' and patients' experiences of the trial's recruitment procedures and	Secondary	1 - GOT-IT (Obstetrics)	Interviews	**27** 10 Obstetricians 6 Clinical midwives	During recruitment period

		balancing hope and uncertainty		consent pathway to see if these aspects of trial delivery could be improved prior to rollout of the main trial…The analysis reported below focuses on staff members' views and experiences of recruiting to the trial" [p.3-4]				11 Research midwives	
Hamdy, F., et al (9)	2018	Partial ablation versus radical prostatectomy in intermediate-risk prostate cancer: the PART feasibility RCT	UK	The qualitative research for the PART feasibility RCT "was embedded into the feasibility study with the aim of understanding the recruitment process and to identify clear obstacles and hidden challenges to recruitment." [p.7]	Secondary	1 – PART (Prostate cancer)	Interviews and audio-recorded consultations	**12+64**<br>**Interviews:**<br>11 Recruiting clinicians<br>1 Research Nurse<br>**Audio-recorded consultations:**<br>64	During recruitment period
Hamilton, D., et al (10)	2013	The recruitment of patients to trials in head and neck cancer: a qualitative study of the EaStER trial of treatments for early laryngeal cancer	UK	"We aimed to investigate the factors contributing to poor recruitment to the EaStER trial." [p.2333]	Secondary	1 – EaStER (Early laryngeal cancer)	Audio-recorded consultations, interviews and focus groups	**6**<br>3 Surgeons<br>3 Recruiters<br>(NB. Number of interviews, focus groups and audio-recordings not reported individually)	Unclear/during recruitment period
Hamlet, C., et al (11)	2017	Recruiting young people with a visible difference to the YP Face IT feasibility trial: a qualitative exploration of primary care staff experiences	UK	"This study aimed to explore GP and nurses' experiences of recruiting to a trial exploring the feasibility of evaluating YP Face IT, a novel online psychosocial intervention to support young people with appearance-altering conditions." [p.541]	Primary	1 - YPF feasibility trial (Online support for visible differences)	Interviews and focus groups	**14**<br>11 GPs<br>3 Nurses	Both during and after recruitment period
Hange, D., et al (12)	2015	Experiences of staff members participating in primary care research activities: a qualitative study	Sweden	"The aim of this paper was to study primary care staff members' experiences and perceptions of participating in a randomized controlled trial concerning Internet therapy." [p.143]	Primary	1-PRIM-NET (Primary Health Care-Internet)	Interviews and focus groups	**34**<br>15 Nurses<br>3 Practice managers<br>3 Study nurses<br>13 GPs	Unclear/during recruitment period

Understanding the perspectives of recruiters is key to improving randomised controlled trial enrolment: a qualitative evidence synthesis – Additional File 7

Hanson, L., et al (13)	2014	Strategies to support recruitment of patient with life-limiting illness for research: the Palliative Care Research Cooperative (PCRC) Group	USA	The objective of this study was “to describe barriers and strategies for recruitment during the inaugural PCRC clinical trial.” [p.1021]	Palliative	1 - not named but described (Statin use in palliative population)	Interviews	**18**<br>PIs and Clinical Research Coordinators (CRCs)	As recruitment period ended
Holm, M., et al (14)	2017	Recruiting participants to a randomized controlled trial testing an intervention in palliative cancer care – the perspectives of health care professionals	Sweden	“The aim of this study is to describe how healthcare professionals experienced recruiting participants to an RCT testing an intervention for family caregivers in specialized palliative cancer care.” [p.7]	Palliative	1 - not named but described (A psycho-educational group intervention trial for family caregivers)	Interviews and focus groups	**25**<br>16 Nurses<br>4 Physicians<br>5 Social workers	Both during and after recruitment period
Howard, L., et al (15)	2009	Why is recruitment to trials difficult? An investigation into recruitment difficulties in an RCT of supported employment in patients with severe mental illness	UK	This study aimed “to evaluate reasons for under-recruitment in an RCT of patients with severe mental illness.” [p.40]	Community/mental health	1 – SWAN (Severe mental illness)	Interviews and workshops	**6 + 17**<br>**Interviews:**<br>1 CI<br>1 PI<br>1 Trial coordinator<br>1 Research nurse<br>2 Care coordinators<br>**Workshops:**<br>17 Care coordinators	During/end of recruitment period
Langley, C., et al (16)	2000	Clinicians' attitudes to recruitment to randomised trials in cancer care: a qualitative study	UK	“This study aimed to examine attitudes to, and difficulties with recruitment to, randomised trials in cancer care by using a combination of a mailed questionnaire and qualitative interviews.” [p.164]	Secondary	Not reported	Interviews	**20**<br>20 Hospital clinicians	Unclear – likely to be retrospective
Mason, V., et al (17)	2007	GPs’ experiences of primary care mental health research: a qualitative study of the barriers to recruitment	UK	“The aim of this study was to investigate the views of GPs towards recruiting patients presenting with depression into RCTs during primary care consultations, in order to identify	Primary	1 – GenPod (Depression)	Interview	**41**<br>41 GPs	Unclear/during recruitment period

				the particular barriers to recruiting this patient group." [p.519					
Paleri, V., et al (18)	2018	Gastrostomy versus nasogastric tube feeding for chemoradiation patients with head and neck cancer: the TUBE pilot RCT	UK	"The aims of this qualitative sub-study of the TUBE trial were to identify, describe and understand (1) barriers to, and facilitators of, trial recruitment; (2) issues of trial conduct; and (3) experiences of feeding tubes." [p.44]	Secondary	1 – TUBE (Head and neck cancer)	Interviews and audio-recorded consultations	**19 + 4**<br>**Interviews:**<br>6 Doctors<br>5 Research nurses<br>5 Dietitians<br>3 SALTs<br>**Audio-recorded consultations:**<br>4	Unclear/during recruitment period
Paramasivan, S., et al (19)	2011	Key issues in recruitment to randomised controlled trials with very different interventions: a qualitative investigation of recruitment to the SPARE trial (CRUK/07/011)	UK	The qualitative research in the SPARE (Selective bladder Preservation Against Radical Excision) feasibility study was undertaken "to explore reasons for low recruitment and attempt to improve recruitment rates by implementing changes suggested by qualitative findings." [p.1]	Secondary	1 -SPARE (Bladder cancer)	Interviews and audio-recorded consultations	**9 + 4**<br>**Interviews**:<br>9 Recruiters<br>**Audio-recorded consultations:**<br>4	During recruitment period
Phelps, E., et al (20)	2019	Facilitating trial recruitment: A qualitative study of patient and staff experiences of an orthopaedic trauma trial	UK	"This study aimed to understand patients' and staffs' experiences of an orthopaedic trauma trial." [p.1] "All staff were asked about their experience of i) recruiting to TrAFFix and other trauma trials, ii) their experience of explaining research studies to patients and iii) their views on their colleagues' engagement with research and TrAFFix" [p.3]	Secondary	1- TrAFFix (Orthopaedics)	Interviews	**24**<br>10 Surgeons<br>14 research associates	Towards end of study
Potter, R., et al (21)	2009	A qualitative study exploring practice nurses' experience of participating in a primary care–based randomised controlled trial	UK	"The aim of this study is to explore the views of practice nurses' recruiting into a primary care–based randomised controlled trial, and to investigate factors that influence the success of trial recruitment." [p.439]	Primary	1 - not named but described (Telephone support for diabetics)	Interviews	**10**<br>10 Practice nurses	Shortly after the recruitment period

Shaheed, C., et al (22)	2014	Participation of pharmacists in clinical trial recruitment for low back pain	Australia	"This study aimed to explore the experiences of pharmacists recruiting patients to a low back pain (LBP) clinical trial conducted in Australia, the challenges they experienced and screening and management of people with acute LBP." [p.986]	Pharmacy	1 – PACE (Lower back pain)	Open ended questionnaire	**30**<br>15 Successful pharmacist recruiters<br>15 Unsuccessful pharmacist recruiters	Unclear
Sin, J., et al (23)	2016	What factors influence successful recruitment of siblings of individuals with first episode psychosis to e- health interventions? A qualitative study	UK	"This study investigated the challenges experienced and strategies employed in the recruitment of siblings of people with first episode psychosis using Early Intervention in Psychosis Services (EIPS) in England." [p.696]	Early Intervention in Psychosis Services (EIPS)	1 - E Sibling Project Randomised Control Trial (E-health intervention for siblings)	Interviews	**12**<br>4 PIs<br>4 Research assistants (clinical studies officer/clinical studies assistant)<br>4 Clinicians	During recruitment period
Skea, Z., et al (24)	2017	'It's trying to manage the work': a qualitative evaluation of recruitment processes within a UK multicentre trial	UK	The aim of the qualitative research integrated into the TISU RCT was "to identify trial-specific modifiable factors that could enhance the facilitators and remove the barriers to recruitment." [p.2]	Secondary	1 – TISU (Ureteric stones)	Interviews	**11**<br>1 Grant co-applicant<br>3 PIs<br>1 Consultant urologist<br>4 Research nurses<br>1 Research assistant	RCT ongoing
Stein, R., et al (25)	2016	OPTIMA prelim: a randomised feasibility study of personalised care in the treatment of women with early breast cancer	UK	The integrated recruitment study in OPTIMA prelim "aimed to work with the chief investigator (CI), TMG and OPTIMA staff to identify and address barriers to recruitment, in three phases." [p.24]	Secondary	1 - OPTIMA Prelim (Breast cancer)	Interviews and audio-recorded consultations	**13 + 36**<br>**Interviews**:<br>8 Oncologists<br>4 Research nurses<br>1 Surgeon<br>**Audio-recorded consultations:**<br>36 oncology appointments	During recruitment period
Strong, S., et al (26)	2016	'The trial is owned by the team, not by an individual': a qualitative	UK	"The aim of this study was to explore how teamwork influences recruitment to a multicentre	Secondary	1 - not named but described	Interviews	**21**<br>8 Surgeons<br>5 Oncologists	During recruitment period

		study exploring the role of teamwork in recruitment to randomised controlled trials in surgical oncology		randomised controlled trial (RCT) involving interventions delivered by different clinical specialties." [p.1]		(Oesophageal cancer)		5 Research nurses<br>1 Specialist nurse<br>1 Research fellow<br>1 Trial coordinator	
Team, V., et al (27)	2018	Barriers and facilitators to participant recruitment to randomised controlled trials: A qualitative perspective	UK	"The aim of this qualitative project was to identify barriers and facilitators to participant recruitment for a randomised double-blinded placebo-controlled trial on the clinical effectiveness of aspirin as an adjunct to compression therapy in healing chronic venous leg ulcers." [p.929]	Primary	1 – ASPiVLU (Venous leg ulcers)	Interviews	**22**<br>4 Wound clinic consultants<br>3 GPs<br>9 Wound clinic nurses<br>4 Research nurses<br>2 Research officers	Unclear/during recruitment period
Tomlin, Z., et al (28)	2012	Patient advocacy and patient centredness in participant recruitment to randomized-controlled trials: implications for informed consent	UK	"This article reports from a study of trial recruitment."[p.672] The objective of the research was "to explore the views and experiences of nurses recruiting patients to randomized-controlled trials and to examine the extent to which their recruitment practices were patient-centred and patient empowering." [p.670]	Secondary	5 – described but not named (Cancer x3, childhood fever, mental health)	Interviews, audio-recorded consultations and group discussion sessions	**9+32+23**<br>**Interviews:**<br>9 Nurses<br>**Group sessions:**<br>30-32 Nurses<br>**Audio-recorded consultations:**<br>23	Following the last audio-recording (during recruitment?)
Wright, J., et al (29)	2002	Factors that Influence the Recruitment of Patients to Phase III Studies in Oncology: The Perspective of the Clinical Research Associate (CRA)	Canada	"The current study was initiated to provide a broader understanding of the factors that may influence patients' decisions regarding entry into randomized, Phase III clinical trials. This understanding was sought by exploring the factors that influence recruitment from the perspective of the CRA and specifically to discuss the impact of	Regional cancer centre	Phase 3 studies	Focus groups	**13**<br>10 Nurses<br>3 Data managers	Unclear/after recruitment experience

				the CRA on the success of patient recruitment.” [p.1585]					
Ziebland, S., et al (30)	2007	Does it matter if clinicians recruiting for a trial don't understand what the trial is really about? Qualitative study of surgeons' experiences of participation in a pragmatic multi-centre RCT	UK	“We used in-depth interviews with surgeons who participated in the Spine Stabilisation Trial (a pragmatic RCT) to explore their understanding of the trial purpose and how this understanding had influenced their recruitment procedures and interpretation of the results.” [p.1]	Secondary	1-Spine Stabilisation Trial (Chronic low back pain)	Interview	**11** Surgeons	Post recruitment period

1. Campbell MK, Snowdon C, Francis D, Elbourne D, McDonald AM, Knight R, et al. Recruitment to randomised trials: strategies for trial enrolment and participation study. The STEPS study. Health Technol Asses. 2007;11(48):113.
2. Clement C, Selman LE, Kehoe PG, Howden B, Lane JA, Horwood J. Challenges to and facilitators of recruitment to an Alzheimer's disease clinical trial: A qualitative interview study. Journal of Alzheimer's Disease. 2019;69(4):1067-75.
3. Donovan JL, Paramasivan S, de Salis I, Toerien M. Clear obstacles and hidden challenges: understanding recruiter perspectives in six pragmatic randomised controlled trials. Trials. 2014;15.
4. Donovan JL, de Salis I, Toerien M, Paramasivan S, Hamdy FC, Blazeby JM. The intellectual challenges and emotional consequences of equipoise contributed to the fragility of recruitment in six randomized controlled trials. J Clin Epidemiol. 2014;67(8):912-20.
5. Ekambareshwar M, Mihrshahi S, Wen LM, Taki S, Bennett G, Baur LA, et al. Facilitators and challenges in recruiting pregnant women to an infant obesity prevention programme delivered via telephone calls or text messages. Trials. 2018;19:9.
6. Frayne SM, Mancuso M, Prout MN, Freund KM. Attitudes of primary care physicians toward cancer-prevention trials: A focus group analysis. Journal of the National Medical Association. 2001;93(11):450-7.
7. Griffin D, Wall P, Realpe A, Adams A, Parsons N, Hobson R, et al. UK FASHIoN: feasibility study of a randomised controlled trial of arthroscopic surgery for hip impingement compared with best conservative care. Health technology assessment (Winchester, England). 2016;20(32):1-172.
8. Hallowell N, Snowdon C, Morrow S, Norman JE, Denison FC, Lawton J. The role of therapeutic optimism in recruitment to a clinical trial in a peripartum setting: balancing hope and uncertainty. Trials. 2016;17:13.
9. Hamdy FC, Elliott D, Le Conte S, Davies LC, Burns RM, Thomson C, et al. Partial ablation versus radical prostatectomy in intermediate-risk prostate cancer: The PART feasibility RCT. Health Technol Assess. 2018;22(52):1-95.
10. Hamilton DW, De Salis I, Donovan JL, Birchall M. The recruitment of patients to trials in head and neck cancer: A qualitative study of the EaStER trial of treatments for early laryngeal cancer. European Archives of Oto-Rhino-Laryngology. 2013;270(8):2333-7.
11. Hamlet C, Williamson H, Harcourt D. Recruiting young people with a visible difference to the YP Face IT feasibility trial: A qualitative exploration of primary care staff experiences. Primary Health Care Research and Development. 2017;18(6):541-8.
12. Hange D, Bjorkelund C, Svenningsson I, Kivi M, Eriksson MC, Petersson EL. Experiences of staff members participating in primary care research activities: A qualitative study. International Journal of General Medicine. 2015;8:143-8.
13. Hanson LC, Bull J, Wessell K, Massie L, Bennett RE, Kutner JS, et al. Strategies to Support Recruitment of Patients With Life-Limiting Illness for Research: The Palliative Care Research Cooperative Group. J Pain Symptom Manage. 2014;48(6):1021-30.
14. Holm M, Alvariza A, Fürst C-J, Wengström Y, Årestedt K, Öhlen J, et al. Recruiting participants to a randomized controlled trial testing an intervention in palliative cancer care – The perspectives of health care professionals. European Journal of Oncology Nursing. 2017;31:6-11.
15. Howard L, de Salis I, Tomlin Z, Thornicroft G, Donovan J. Why is recruitment to trials difficult? An investigation into recruitment difficulties in an RCT of supported employment in patients with severe mental illness. Contemp Clin Trials. 2009;30(1):40-6.
16. Langley C, Gray S, Selley S, Bowie C, Price C. Clinicians' attitudes to recruitment to randomised trials in cancer care: a qualitative study. Journal of health services research & policy. 2000;5(3):164-9.
17. Mason VL, Shaw A, Wiles NJ, Mulligan J, Peters TJ, Sharp D, et al. GPs' experiences of primary care mental health research: a qualitative study of the barriers to recruitment. Fam Pract. 2007;24(5):518-25.
18. Paleri V, Patterson J, Rousseau N, Moloney E, Craig D, Tzelis D, et al. Gastrostomy versus nasogastric tube feeding for chemoradiation patients with head and neck cancer: The tube pilot RCT. Health Technol Assess. 2018;22(16).
19. Paramasivan S, Huddart R, Hall E, Lewis R, Birtle A, Donovan JL. Key issues in recruitment to randomised controlled trials with very different interventions: a qualitative investigation of recruitment to the SPARE trial (CRUK/07/011). Trials. 2011;12:15.
20. Phelps EE, Tutton E, Griffin X, Baird J, Costa ML, Parsons N, et al. Facilitating trial recruitment: A qualitative study of patient and staff experiences of an orthopaedic trauma trial. Trials. 2019;20(1):492.
21. Potter R, Dale J, Caramlau I. A qualitative study exploring practice nurses' experience of participating in a primary care-based randomised controlled trial. Journal of Research in Nursing. 2009;14(5):439-47.
22. Shaheed CA, Maher CG, Williams KA, McLachlan AJ. Participation of pharmacists in clinical trial recruitment for low back pain. International Journal of Clinical Pharmacy. 2014;36(5):986-94.
[illegible]
