# Supplementary material for: Understanding the perspectives of recruiters is key to improving randomised controlled trial enrolment: a qualitative evidence synthesis
Source: Trials. 2022 Oct 20;23:883. doi: 10.1186/s13063-022-06818-4 (PMC9585862; doi:10.1186/s13063-022-06818-4)
Supplement: Supplementary file 8 — Additional file 8. Recruiter Roles. Outline of the roles of the recruiters included in the synthesis. [file 13063_2022_6818_MOESM8_ESM.pdf]

Understanding the perspectives of recruiters is key to improving randomised controlled trial enrolment: a qualitative evidence synthesis – Additional File 8

Listed professions of recruiters included in the synthesis, and the studies in which they were reported

- Research nurses (1-7)
- Nurses (8-13)
- Surgeons (5, 6, 14-17)
- General Practitioners (GPs) (10, 14, 18, 19)
- Principal investigators (PIs) (2, 4, 20-22)
- Doctors (3, 8, 9)
- Oncologist (5, 6, 14)
- Recruiters (15, 23, 24)
- Chief investigators (CIs) (2, 9)
- Clinicians (22, 25)
- Physicians (11, 26)
- Research assistants (4, 22)
- Research associates (16, 25)
- Trial coordinators (2, 6)
- Care coordinators (2)
- Central recruiters (20)
- Clinical midwives (27)
- Clinical research coordinators (21)
- Community mental health staff (14)
- Consultant urologist (4)
- Data managers (13)
- Dietitians (3)
- Grant co-applicant (4)
- Hospital clinicians (28)
- Local lead investigators (20)
- Obstetricians (27)
- Pharmacist (29)
- Practice managers (10)
- Practice nurses (30)
- Recruiting clinicians (1)

- Recruiting doctors (20)
- Recruiting nurses (20)
- Research fellow (6)
- Research midwives (27)
- Research officer (7)
- Speech and language therapists (SALTs)(3)
- Social workers (11)
- Specialist nurse (6)
- Study nurses (10)
- Trial Management Group (9)
- Wound clinic consultants (7)
- Wound clinic nurses (7)

1. Hamdy FC, Elliott D, Le Conte S, Davies LC, Burns RM, Thomson C, et al. Partial ablation versus radical prostatectomy in intermediate-risk prostate cancer: The PART feasibility RCT. Health Technol Assess. 2018;22(52):1-95.
2. Howard L, de Salis I, Tomlin Z, Thornicroft G, Donovan J. Why is recruitment to trials difficult? An investigation into recruitment difficulties in an RCT of supported employment in patients with severe mental illness. Contemp Clin Trials. 2009;30(1):40-6.
3. Paleri V, Patterson J, Rousseau N, Moloney E, Craig D, Tzelis D, et al. Gastrostomy versus nasogastric tube feeding for chemoradiation patients with head and neck cancer: The tube pilot RCT. Health Technol Assess. 2018;22(16).
4. Skea ZC, Treweek S, Gillies K. It's trying to manage the work': A qualitative evaluation of recruitment processes within a UK multicentre trial. Bmj Open. 2017;7(8):e016475.
5. Stein RC, Dunn JA, Bartlett JMS, Campbell AF, Marshall A, Hall P, et al. OPTIMA prelim: a randomised feasibility study of personalised care in the treatment of women with early breast cancer. Health technology assessment (Winchester, England). 2016;20(10):xxiii-201.
6. Strong S, Paramasivan S, Mills N, Wilson C, Donovan JL, Blazeby JM. 'The trial is owned by the team, not by an individual': a qualitative study exploring the role of teamwork in recruitment to randomised controlled trials in surgical oncology. Trials. 2016;17:1-9.
7. Team V, Bugeja L, Weller CD. Barriers and facilitators to participant recruitment to randomised controlled trials: A qualitative perspective. International Wound Journal. 2018;15(6):929-42.
8. Clement C, Selman LE, Kehoe PG, Howden B, Lane JA, Horwood J. Challenges to and facilitators of recruitment to an Alzheimer's disease clinical trial: A qualitative interview study. Journal of Alzheimer's Disease. 2019;69(4):1067-75.
9. Donovan JL, Paramasivan S, de Salis I, Toerien M. Clear obstacles and hidden challenges: understanding recruiter perspectives in six pragmatic randomised controlled trials. Trials. 2014;15.

10. Hange D, Bjorkelund C, Svenningsson I, Kivi M, Eriksson MC, Petersson EL. Experiences of staff members participating in primary care research activities: A qualitative study. International Journal of General Medicine. 2015;8:143-8.
11. Holm M, Alvariza A, Fürst C-J, Wengström Y, Årestedt K, Öhlen J, et al. Recruiting participants to a randomized controlled trial testing an intervention in palliative cancer care – The perspectives of health care professionals. European Journal of Oncology Nursing. 2017;31:6-11.
12. Tomlin Z, deSalis I, Toerien M, Donovan JL. Patient advocacy and patient centredness in participant recruitment to randomized-controlled trials: implications for informed consent. Health Expectations. 2014;17(5):670-82.
13. Wright JR, Crooks D, Ellis PM, Mings D, Whelan TJ. Factors that influence the recruitment of patients to phase III studies in oncology - The perspective of the clinical research associate. Cancer. 2002;95(7):1584-91.
14. Donovan JL, de Salis I, Toerien M, Paramasivan S, Hamdy FC, Blazeby JM. The intellectual challenges and emotional consequences of equipoise contributed to the fragility of recruitment in six randomized controlled trials. J Clin Epidemiol. 2014;67(8):912-20.
15. Hamilton DW, De Salis I, Donovan JL, Birchall M. The recruitment of patients to trials in head and neck cancer: A qualitative study of the EaStER trial of treatments for early laryngeal cancer. European Archives of Oto-Rhino-Laryngology. 2013;270(8):2333-7.
16. Phelps EE, Tutton E, Griffin X, Baird J, Costa ML, Parsons N, et al. Facilitating trial recruitment: A qualitative study of patient and staff experiences of an orthopaedic trauma trial. Trials. 2019;20(1):492.
17. Ziebland S, Featherstone K, Snowdon C, Barker K, Frost H, Fairbank J. Does it matter if clinicians recruiting for a trial don't understand what the trial is really about? Qualitative study of surgeons' experiences of participation in a pragmatic multi-centre RCT. Trials. 2007;8:4.
18. Hamlet C, Williamson H, Harcourt D. Recruiting young people with a visible difference to the YP Face IT feasibility trial: A qualitative exploration of primary care staff experiences. Primary Health Care Research and Development. 2017;18(6):541-8.
19. Mason VL, Shaw A, Wiles NJ, Mulligan J, Peters TJ, Sharp D, et al. GPs' experiences of primary care mental health research: a qualitative study of the barriers to recruitment. Fam Pract. 2007;24(5):518-25.
20. Campbell MK, Snowdon C, Francis D, Elbourne D, McDonald AM, Knight R, et al. Recruitment to randomised trials: strategies for trial enrolment and participation study. The STEPS study. Health Technol Asses. 2007;11(48):113.
21. Hanson LC, Bull J, Wessell K, Massie L, Bennett RE, Kutner JS, et al. Strategies to Support Recruitment of Patients With Life-Limiting Illness for Research: The Palliative Care Research Cooperative Group. J Pain Symptom Manage. 2014;48(6):1021-30.
22. Sin J, Henderson C, Spain D, Gamble C, Norman I. What factors influence successful recruitment of siblings of individuals with first episode psychosis to e-health interventions? A qualitative study. Health Expectations. 2016.
23. Ekambareshwar M, Mihrshahi S, Wen LM, Taki S, Bennett G, Baur LA, et al. Facilitators and challenges in recruiting pregnant women to an infant obesity prevention programme delivered via telephone calls or text messages. Trials. 2018;19:9.
24. Paramasivan S, Huddart R, Hall E, Lewis R, Birtle A, Donovan JL. Key issues in recruitment to randomised controlled trials with very different interventions: a qualitative investigation of recruitment to the SPARE trial (CRUK/07/011). Trials. 2011;12:15.
25. Griffin D, Wall P, Realpe A, Adams A, Parsons N, Hobson R, et al. UK FASHIoN: feasibility study of a randomised controlled trial of arthroscopic surgery for hip impingement compared with best conservative care. Health technology assessment (Winchester, England). 2016;20(32):1-172.

26. Frayne SM, Mancuso M, Prout MN, Freund KM. Attitudes of primary care physicians toward cancer-prevention trials: A focus group analysis. Journal of the National Medical Association. 2001;93(11):450-7.
27. Hallowell N, Snowdon C, Morrow S, Norman JE, Denison FC, Lawton J. The role of therapeutic optimism in recruitment to a clinical trial in a peripartum setting: balancing hope and uncertainty. Trials. 2016;17:13.
28. Langley C, Gray S, Selley S, Bowie C, Price C. Clinicians' attitudes to recruitment to randomised trials in cancer care: a qualitative study. Journal of health services research & policy. 2000;5(3):164-9.
29. Shaheed CA, Maher CG, Williams KA, McLachlan AJ. Participation of pharmacists in clinical trial recruitment for low back pain. International Journal of Clinical Pharmacy. 2014;36(5):986-94.
30. Potter R, Dale J, Caramlau I. A qualitative study exploring practice nurses' experience of participating in a primary care-based randomised controlled trial. Journal of Research in Nursing. 2009;14(5):439-47.
